# *Aplf/Dna2* variants drive chromosomal fission and accelerate speciation in zokors

**DOI:** 10.1126/sciadv.adt2282

**Published:** 2025-09-05

**Authors:** Na Wan, Qijiao Duan, Zhenyuan Cai, Zhanwu Zhu, JingOu Wang, Yonghui Tian, Wei Shen, Bowen Li, Zhuoran Kuang, Xiaolong Liang, Sanyuan Liu, Xuan An, Xiaojie Yang, Xi Liu, Leyan Mao, Jiaqi Chen, Yinjia Wang, Zhilong Feng, Wenwen Liu, Yueting Bu, Eviatar Nevo, Riccardo Papa, Axel Meyer, Jianquan Liu, Kexin Li

**Affiliations:** ^1^ State Key Laboratory of Herbage Improvement and Grassland Agro-Ecosystems, College of Ecology, Lanzhou University, Lanzhou, P. R. China.; ^2^ Key Laboratory of Adaptation and Evolution of Plateau Biota, Northwest Institute of Plateau Biology, Chinese Academy of Sciences, Xining 810001, China.; ^3^ School of Life Sciences, Lanzhou University, Lanzhou 730000, China.; ^4^ School of Life Sciences, Zhengzhou University, Zhengzhou, Henan Province 450001, China.; ^5^ The Fifth High School of Gaomi City, Gaomi, Shandong Province, 261500, China.; ^6^ Institute of Evolution, University of Haifa, Haifa 3498838, Israel.; ^7^ Department of Biology, University of Puerto Rico at Río Piedras, San Juan 00931, Puerto Rico.; ^8^ Molecular Sciences and Research Center, University of Puerto Rico, San Juan 00931, Puerto Rico.; ^9^ Dipartimento di Scienze Chimiche della Vita e della Sostenibilità Ambientale, Università di Parma, Parma, 43121, Italy.; ^10^ Department of Biology, University of Konstanz, Konstanz, Germany.; ^11^ Museum of Comparative Zoology, Harvard University, Cambridge MA 02138, USA.; ^12^ South China Sea Institute of Oceanology, Chinese Academy of Sciences, No. 164, Xingangxi Rd, Guangzhou, China.

## Abstract

Chromosomal fissions and fusions are common, yet the molecular mechanisms and implications in speciation remain poorly understood. Here, we confirm a fission event in one zokor species through multiple-omics and functional analyses. We traced this event to a mutation in a splicing enhancer of the DNA repair gene *Aplf* in the fission-bearing species, which caused exon skipping and produced a truncated protein that disrupted DNA repair. An intronic deletion in *Dna2*, known to facilitate neo-telomere formation when knocked out, reduced gene activity. These variants collectively drove chromosomal fission in this zokor species. The newly formed chromosome became fixed due to carrying essential genes and strong selective pressure. While geographic isolation likely initiated the divergence of this species and the sister one, the fission event and associated decline at the chromosome level in gene flow probably exacerbated the speciation process. Our work elucidates the genetic basis of chromosomal fission and underscores its role in speciation dynamics.

## Teaser

*Aplf* splicing defect and *Dna2* deletion drove chromosomal fission, potentially exacerbating speciation divergence in zokors.

## INTRODUCTION

Chromosomal rearrangements, including fissions or fusions, are widespread across the animal kingdom (*1*–*7*). These changes can be observed from fishes (*4*) and reptiles (*3*) to birds (*3*, *8*) to mice (*9*), and to some subterranean mammals such as blind mole rat (*1*), *Fukomys* (*10*), and *Cryptomys* (*10*). Chromosomal rearrangements have profoundly shaped species diversity by altering genomic architecture. However, mechanistic drivers of chromosomal fission and their effects on speciation remain poorly understood.

Centric fission, the division of a chromosome at its centromere, is rare compared to fusion (*11*). The centromere, comprising a large tandem satellite repeat array, is inherently fragile and prone to DNA breaks (*12*). When double-strand breaks (DSBs) occur, and if DNA damage repair fails, telomerase adds telomeric repeats to DSBs, leading to interstitial telomeric repeat insertions that can serve as seeds for telomere addition (*13*) or formation of functional neo-telomeres accompanied by terminal deletions (*14*). Aprataxin-and-pnk-like factor (*Aplf*) is crucial for DNA repair via nonhomologous end joining (NHEJ) (*15*), and it encodes a protein with two functional domains: a forkhead-associated (FHA) domain (exons 1 to 3) that interacts with DNA repair proteins x-ray repair cross complementing 4 (XRCC4) and x-ray repair cross complementing 1 (XRCC1) and two PAR-binding zinc finger (PBZ) domains (exons 7 to 9) that recruit APLF protein to damage sites (*16*). The truncation of either domain could impair NHEJ (*17*) and delay DSB repair, which may lead to neo-telomere formation (*18*). Suppressing DNA repair genes such as DNA replication helicase/nuclease 2 (*Dna2*) and exonuclease 1 (*Exo1*) was shown to enhance functional neo-telomere formation (*14*) and exacerbate chromosomal fission (*8*). While chromosomal fissions risk deleterious gene loss, their fixation depends on fitness effects, driven by selection advantages or genetic drift (*19*, *20*). However, how these mutations interact with geographic isolation to catalyze speciation remains unclear (*20*).

**Fig. 1.**
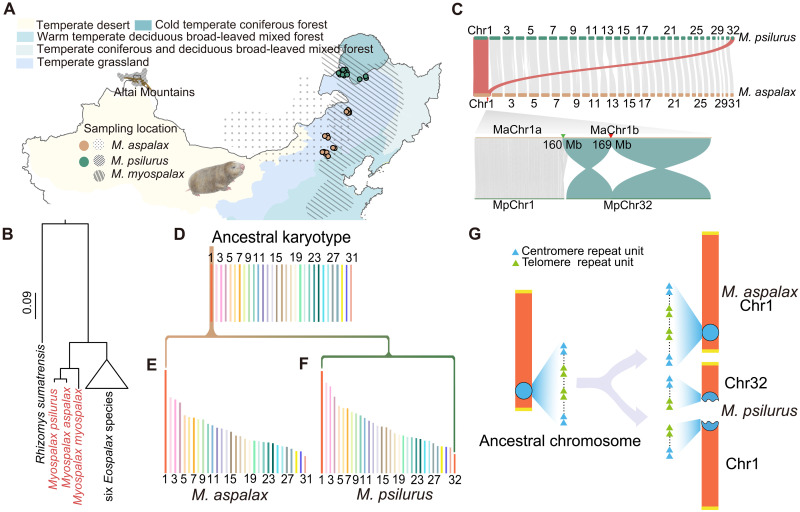
Study species and chromosomal fission. (**A**) Distribution of *M. aspalax*, *M. psilurus*, and *M. myospalax*. Green and brown circles represent sample locations for *M. aspalax* and *M. psilurus*, respectively. Gray dots and gray lines shading, respectively, indicate the distribution of the three species based on published literature (*31*), with different color shades representing various climate types. (**B**) Phylogenetic tree showing the evolutionary relationships among all nine Myospalacinae species based on mitochondrial cytochrome *b* gene sequences. *R. sumatrensis* was used as the outgroup to root the tree. (**C**) Top: Chromosomal synteny analysis between the genomes of *M. aspalax* and *M. psilurus*. Notably, *M. aspalax* chromosome 1 (MaChr1) shows synteny with both chromosome 1 (MpChr1) and chromosome 32 of *M. psilurus* (MpChr32). Bottom: Syntenic blocks in the local fission region between *MaChr1* and *MpChr1 and MpChr32*. MaChr1a corresponds to Chr1: 1 to 160 Mb of *M. aspalax*, while MaChr1b spans 160 to 193.8 Mb. The collinearity block between MaChr1 and MpChr32 revealed two inversions (green blocks) with breakpoints at 160 Mb (green arrow) and 169 Mb (red arrow). (**D**) Reconstructed ancestral chromosome segments for *M. aspalax* and *M. psilurus*. Each colored column represents a distinct protosegment, with the numbers below indicating the corresponding ancestral chromosome. (**E**) Distribution of ancestral chromosome segments in the genome of *M. aspalax*, showing that segments derived from proto-chromosome 1 were retained on chromosome 1. (**F**) Distribution of ancestral chromosome segments in the genome of *M. psilurus*, where segments originating from proto-chromosome 1 were dispersed across chromosomes 1 and 32. (**G**) Schematic representation showing that chromosomes 1 and 32 in *M. psilurus* originated from a centromeric fission event, while chromosome 1 in *M. aspalax* retained the ancestral state. The blue circle represents centromeres, and the blue and green triangles indicate centromere and telomere repeat units at the centromere.

Chromosomal fission and fusion may drive population divergence by inducing meiotic errors (e.g., aneuploidy and unsynapsed homologs) that decrease hybrid fertility (*21*, *22*). These mismatches can reduce recombination and amplify genomic divergence, potentially acting as one of the major reproductive barriers (*5*, *23*). While theoretical models posit that suppressed gene flow near fission/fusion breakpoints and/or fused chromosome(s) or neo-chromosomes accelerate interspecific differentiation (*24*, *25*), empirical evidence remains conflicting: Some sibling species with distinct karyotypes interbreed frequently without total reproductive isolation, underscoring unresolved complexities in chromosomal speciation (*26*, *27*). Here, we used zokors (subfamily Myospalacinae, family Spalacidae), subterranean rodents endemic to North China (*28*), to address these questions. Their hypoxic burrow environments impose strong selective pressures on efficient DNA repair-a trait critical for mitigating oxidative damage (*29*, *30*). Among zokors, *Myospalax aspalax* (*2n* = 62) and *Myospalax psilurus* (*2n* = 64) are sister species primarily distributed in different climatic regions (fig. S1A and data S1) yet with overlapping ranges, contrasting with the karyotypically distinct *Myospalax myospalax* (*2n* = 44) isolated in the Altai Mountains (*31*) (Fig. 1A). This divergence, coupled with their adaptation to hypoxia and variable karyotypes, positions *M. aspalax* and *M. psilurus* as an ideal model to dissect chromosomal speciation mechanisms.

In this study, we integrated de novo chromosome-level assemblies, collinearity mapping, and three-dimensional (3D) genome analyses to pinpoint one chromosomal fission event in *M. psilurus*. Long-read sequencing and CRISPR-Cas9 editing reveal how splicing defects in *Aplf* and *Dna2* deletions disrupt DNA repair, driving fission in this species. Population genomic data further clarify how reduced gene flow and selection facilitated fixation of this karyotype, linking chromosomal rearrangement to reproductive isolation between it and the sister species in addition to geographic isolation.

## RESULTS

### De novo genome assembly and annotation

We constructed a phylogenetic tree for all nine Myospalacinae species based on the cytochrome *b* gene, with *Rhizomys sumatrensis* as the outgroup, confirming *M. psilurus* and *M. aspalax* as sister species (Fig. 1B). To identify chromosomal rearrangements between the two sibling species, we conducted de novo genome assemblies using 83.87 and 96.01 Gb of PacBio HiFi reads (tables S1 to S3). Final genome assembly sizes for *M. aspalax* and *M. psilurus* were 3.02 Gb (mMyoAsp6.1) and 3.31 Gb (mMyoPsi5.1) (table S4), with heterozygosity of 0.29 and 0.22%, respectively (fig. S1, B and C). The Contig N50 sizes were 37.95 and 23.28 Mb for *M. aspalax* and *M. psilurus*, respectively (table S4). Benchmarking Universal Single-Copy Orthologs (BUSCO) analysis indicated 96.6 and 96.7% completeness for *M. aspalax* and *M. psilurus*, respectively (fig. S1D and tables S5 and S6). The genome-wide guanine-cytosine (GC) content for *M. aspalax* and *M. psilurus* was 41.49 and 41.72%, respectively (fig. S1E). Using high-throughput chromatin conformation (Hi-C) data (~103.13× for *M. aspalax* and ~92.28× for *M. psilurus*; tables S7 and S8), we anchored the contigs onto 31 and 32 pseudo-chromosomes, respectively, consistent with previously reported karyotypes (fig. S1, F and G, and tables S9 and S10) (*32*).

We predicted 21,060 and 21,004 protein-coding genes for *M. aspalax* and *M. psilurus* respectively, supported by homology searches, transcriptome evidence, and ab initio predictions. BUSCO analyses showed 92.4 and 92.6% completeness for the gene annotations (tables S11 and S12). Simple repeat sequences accounted for 8.65% (261.87 Mb) of the *M. aspalax* genome and 18.47% (611.84 Mb) of the *M. psilurus* genome (tables S13 and S14), contributing to the larger genome size of the *M. psilurus*. Notably, the proportion of telomeric sequences in *M. psilurus* (216.13 Mb, 6.52%) was substantially higher than in *M. aspalax* (56.78 Mb, 1.87%). In addition, transposable elements (TEs) constituted 47.59% of the *M. aspalax* genome and 43.57% of the *M. psilurus* genome, including 466.76 Mb (15.41%) and 425.93 Mb (12.86%) of long interspersed nuclear elements in *M. aspalax* and *M. psilurus*, respectively, 461.38 Mb (15.23%) and 420.74 Mb (12.70%) of short interspersed nuclear elements, 438.52 Mb (14.48%) and 530.40 Mb (16.01%) of long terminal repeat retrotransposons (LTR-RTs) and 74.78 Mb (2.47%) and 66.46 Mb (2.01%) of DNA transposons (tables S13 and S14).

### Chromosome synteny analysis and identification of chromosomal fission

Chromosome synteny analysis indicated collinearity between chromosome 1 of *M. aspalax* (MaChr1) and chromosomes 1 and 32 of *M. psilurus* (MpChr1 and MpChr32) (Fig. 1C). To determine whether it is a fission event in *M. psilurus* or a fusion event in *M. aspalax*, we reconstructed the ancestral karyotype of the two species taking *Eospalax fontanierii* (a closely related species belonging to the same subfamily Myospalacinae but a different genus) as an outgroup and obtained 31 (*2n* = 62) proto-chromosomes (Fig. 1D). Proto-chromosome 1 segments mapped to MpChr1 and MpChr32 but only to MaChr1, indicating a fission event in *M. psilurus* (Fig. 1, E and F). To confirm this fission event, we aligned *M. aspalax* and *M. psilurus* with four outgroup species: *E. fontanierii*, *Eospalax cansus*, *Spalax carmeli* (a species from the same family Spalacidae but a different subfamily, Spalacinae), and *Rattus norvegicus* (a species from the same order Rodentia but a different family, Muridae). A phylogenetic tree clearly illustrates the relationships among these species (fig. S2A). Regardless of the alignments with closely related outgroups or distant outgroups, MaChr1 exhibited continuous synteny with outgroups, without any indications of breakage (fig. S2B) (*33*). This further confirmed that the chromosomal fission occurred in the ancestral lineage leading to MpChr1 and MpChr32 (Fig. 1G).

### Centric fission and ancestral ITS

**Fig. 2.**
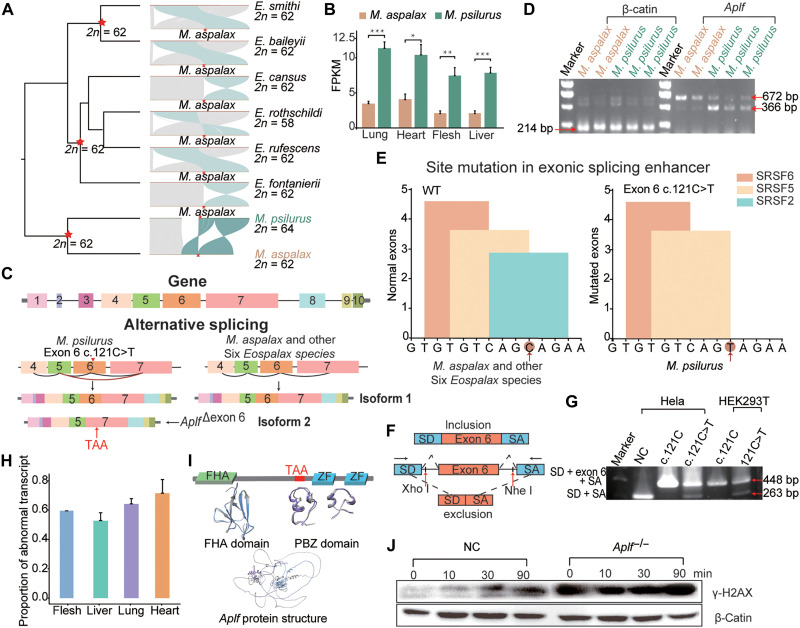
*Aplf* gene disruption and DNA repair deficiency in *M. psilurus*. (**A**) Left: Phylogenetic tree of eight Myospalacinae species. Middle: Collinearity between *M. aspalax* and the other seven Myospalacinae species around the fission breakpoint. Right: Species names and corresponding karyotype numbers. Green blocks indicate frequent rearrangement regions of the eight Myospalacinae species, and red arrows mark large (~2.7 Mb) interstitial telomeric sequence; red stars indicate ancestral karyotypes. (**B**) *Aplf* expression is significantly up-regulated in the lung, heart, flesh and liver of *M. psilurus* compared to *M. aspalax*. (**C**) Full-length transcriptome analysis shows alternative splicing in *M. psilurus*, resulting in exon 6 skipping due to the c.121C>T mutation, generating an aberrant isoform (isoform 2) alongside the normal isoform (isoform 1), whereas *M. aspalax* and *Eospalax* species express only isoform 1. (**D**) PCR validation reveals a 672-bp product for exon 6 inclusion and a 366-bp product for exclusion, with β-actin as the control. (**E**) c.121C>T mutation disrupts a predicted ESE. Regulatory factors SRSF6 (orange), SRSF5 (yellow), and SRSF2 (green) are indicated. (**F**) Schematic of the in vitro minigene assay: exon 6-inclusion transcripts (top) and exon 6-exclusion transcripts (bottom). Arrows indicate PCR primer positions. (**G**) Minigene PCR products in HeLa and HEK293T cells: lanes show marker; NC, empty vector; plasmids with wild-type (c.121C) and mutant (c.121C>T) genotypes. (**H**) Proportion of transcripts with exon 6 skipping among all transcripts of the *Aplf* gene in four tissues. (**I**) Diagram of APLF protein domains: FHA (green) and two PBZ (blue) domains. A premature TAA codon between FHA and PBZ results in loss of PBZ domains in *M. psilurus*. (**J**) Western blot of γH2AX expression in *Aplf* knockout (*Aplf*^−/−^) versus control cells (NC) after DNA damage treatment at 0, 10, 30, and 90 min. β-Actin is the loading control. Statistical significance is indicated as *P < 0.05, **P < 0.01, and ***P < 0.001. SNP, single-nucleotide polymorphism; WT, wild type; FPKM, fragments per kilobase of transcript per million mapped reads.

To find out the precise breakpoint, we performed chromatin immunoprecipitation sequencing (ChIP-Seq) analysis to map the centromeres of MaChr1, MpChr1, and MpChr32 (table S15). The results revealed peak enrichments at ~169 to 171.5 Mb on MaChr1 and near the terminal region of MpChr1 and MpChr32, suggesting a centric fission event (fig. S2, C to E). Based on the unexpected bias clustered substitutions (UBCS) analysis of weak-to-strong (AT→GC) substitutions (*34*) flanking the fission breakpoints in both *M. aspalax* and *M. psilurus*, we estimated that the chromosomal fission event occurred approximately 1.12 million years ago (fig. S2F). The telomere repeat unit (TTAGGG)*n* was enriched at two distinct genomic locations: (1) near the 169 Mb position of MaChr1, and (2) at the terminal regions of MpChr1 and MpChr32 (fig. S2G). The exceptionally large interstitial telomeric sequence (ITS) near the 169-Mb position on MaChr1 spanned ~2.7 Mb (fig. S2G). Intriguingly, the location of the ITS was conserved across eight Myospalacinae species (Fig. 2A), indicating inheritance from a common ancestor of both genera. However, diversified nonsyntenic regions around the ITS suggest that it is a rearrangement hotspot (Fig. 2A).

### *Aplf* gene disruption, causal mechanism, and DNA repair deficiency

The break at the fission breakpoint was observed in eight Myospalacinae species (Fig. 2A), with successful reconnection occurring in seven species except for *M. psilurus*, which experienced a fission event. To investigate the specific failure of reconnection in *M. psilurus*, we adopted a multi-omics approach. First, we conducted differential gene expression analysis using bulk RNA sequencing (bulk RNA-seq) (table S16), identifying 3933 genes with significant expression differences between *M. psilurus* and *M. aspalax* [log_2_(fold change) > 1 and adjusted *P* value < 0.005; fig. S3, A and B]. Of these, 87 and 47 genes showed consistent up- and down-regulation across multiple tissues (lung, heart, muscle, and liver) in *M. psilurus* (fig. S3, A and B). The up-regulated genes were significantly enriched in pathways such as DNA repair and homologous chromosome segregation, whereas the down-regulated genes were predominantly associated with nonsense-mediated decay and translation processes (fig. S3C). Among these, five genes—*Aplf*, synaptonemal complex protein 1 (*Sycp1*), synaptonemal complex protein 3 (*Sycp3*), testis-expressed 15 (*Tex15*), and ring finger protein 169 (*Rnf169*)—linked to DNA damage repair were significantly up-regulated in *M. psilurus* than *M. aspalax* (Fig. 2B and fig. S3C, D). Second, we performed alternative splicing analysis using isoform sequencing data (table S17), revealing 6,157 genes with distinct splicing patterns in *M. psilurus*. When intersecting the five genes exhibiting differential expression and genes with alternative splicing, only *Aplf* and *Tex15* remain. Third, a transcriptome-wide survey of gene loss events based on isoform sequencing data uncovered 270 pseudogenes in *M. psilurus* and 239 in *M. aspalax*. By integrating these datasets—differential expression, splicing variation, and gene loss—we identified *Aplf*, a gene involved in DNA damage repair, as a standout candidate for further investigation. *Aplf* showed two isoforms: isoform 1 containing all 10 exons and isoform 2 with 9 exons due to exon 6 skipping (*Aplf*^∆exon 6^). Only complete isoform 1 was observed in *M. aspalax* (Fig. 2C).

The presence of the *Aplf*^ ∆exon 6^ transcript in *M. psilurus*, absent in *M. aspalax*, was confirmed by reverse transcription polymerase chain reaction (RT-PCR) in multiple individuals, generating two PCR products of different lengths (Fig. 2D). To investigate the cause of the exon 6 skipping splicing in the *Aplf* gene of *M. psilurus*, we analyzed splicing regulatory elements for exon 6 and identified a single-nucleotide mutation of c.121C>T from *M. aspalax* to *M. psilurus*. This mutation disrupts an exonic splicing enhancer (ESE) motif (CAGCAGA>CAGTAGA) that binds with splicing factor serine and arginine rich splicing factor 2 (SRSF2) protein (Fig. 2E), leading to the observed alternative splicing. Notably, this mutation is unique to *M. psilurus* among the 173 individuals from the eight Myospalacinae species we examined (fig. S3E). To validate the role of this mutation in mediating exon 6 skipping, we performed minigenes splicing assay, which contains two cassette exons (SD and SA) within an expression system (Fig. 2F). Results from both human embryonic kidney 293T (HEK293T) cells and HeLa indicated that transfection of exon 6 c.121C plasmid produced a complete transcript with exonSD, inserted exon 6, and exonSA, whereas transfection of exon 6 c.121C>T plasmid additionally generated an exon 6-excluded transcript similar to empty vector (Fig. 2G). In addition, we estimated the fixation time of the site mutation in *Aplf*, and it was ~1.87 million years ago (see Materials and Methods).

Moreover, the abnormal transcript accounted for 50% or more of the total gene expression in four tissues of *M. psilurus*, especially in the heart (Fig. 2H). The exon 6 skipping in *Aplf* caused a frameshift mutation, introducing a premature termination codon (PTC) of TAA in exon 7 (Fig. 2C). This PTC resulted in the absence of PBZ domains in the *Aplf* protein of *M. psilurus* (Fig. 2I). To assess whether *Aplf* gene disruption affected DNA damage repair, we used CRISPR-Cas9 to knock out the *Aplf* gene with single guide RNAs (sgRNAs) targeting exon7 to truncate the PBZ domain of the APLF protein in mouse embryonic fibroblasts. DNA damage–induced assay indicated that under equal treatment times, there were more γH2AX signals in the knockout cells compared to the wild-type cells, indicating a potential functional deficiency of *Aplf* due to the PTC-induced truncation in *M. psilurus* (Fig. 2J).

### Genomic structural variation and neo-telomere formation

**Fig. 3.**
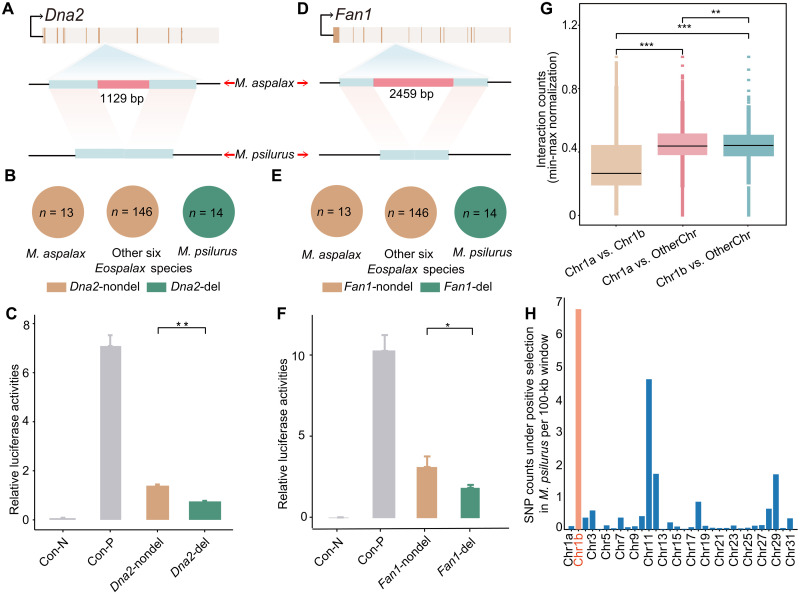
Genomic structural variation of *Fan1* and *Dna2* in *M. psilurus*. (**A**) Schematic exhibiting a 1129-bp intronic deletion in *Dna2* gene of *M. psilurus*. (**B**) Frequency of the *Dna2* intronic deletion across 173 Myospalacinae individuals*.* All 14 *M. psilurus* individuals exhibited the homozygous deletion, while it was absent in 159 individuals of *M. aspalax* and six *Eospalax* species. (**C**) Functional testing of the *Dna2* intronic deletion using a DLRA in HEK293T cells, demonstrating decreased regulatory activity. The pGL3-basic and pGL3-enhancer plasmids served as negative (Con-N) and positive controls (Con-P), respectively. (**D**) A 2459-bp intronic deletion in the *Fan1* gene in *M. psilurus*. (**E**) Frequency of the *Fan1* intronic deletion across 173 individuals, indicating its fixation in *M. psilurus*. (**F**) Functional testing of the *Fan1* deletion using DLRA, showing decreased gene activity in *M. psilurus*. (**G**) Normalized chromatin contact counts between MaChr1a and MaChr1b, between MaChr1a and other nonfissioned chromosomes, and between MaChr1b and other nonfissioned chromosomes. Intrachromosomal interactions within MaChr1 (between MaChr1a and MaChr1b) are significantly fewer than interchromosomal interactions (between MaChr1a or MaChr1b and other nonfissioned chromosomes). (**H**) Selective signals of fissioned chromosome compared to other nonfissioned chromosomes in *M. psilurus*. Chr1a: Chr1: 1 to 160 Mb of *M. aspalax*; Chr1b: 160 to 193.8 Mb of *M. aspalax*. ***P < 0.001, **P < 0.01, and *P < 0.05.

Deficiencies in *Atr*, *Dna2*, and *Exo1* have been linked to neo-telomere formation (*14*). To investigate the formation of functional terminal neo-telomere at the broken ends in *M. psilurus*, we focused on structural variants (SVs) that might disrupt genes involved in telomerase activity, thereby promoting the addition of TTAGGG repeats at DSBs. Using long-read DNA sequencing from two individuals of *M. aspalax*, three individuals of *M. psilurus*, and integrating 17 previously published long-read sequencing data from six *Eospalax* species (table S18), we identified 68,679 SVs larger than 50 base pair (bp), among which, 868 were specific to and fixed in *M. psilurus*. We annotated the genes closest to these SVs and identified 118 genes, which are enriched in DSB repair, DNA damage response, and heart morphogenesis (fig. S3F). Notably, 15 of these genes showed significant enrichment in DSB repair (Fisher’s exact test: *P*-value = 0.00182). Among these 15, Fanconi-associated nuclease 1 (*Fan1*), *Dna2*, and minichromosome maintenance domain containing 2 (*Mcmdc*2) emerged as prime candidates due to their critical roles in maintaining genomic integrity (Fig. 3, A and D, and figs. S4 and S5A). We verified these deletions in 173 zokor individuals from eight species of subfamily Myospalacinae (14 *M. psilurus* and 13 *M. aspalax* individuals from the genus *Myospalax* and 146 individuals across six *Eospalax* species from previous studies), confirming that they were specific and fixed in *M. psilurus* (Fig. 3, B and E; fig. S5B; and table S19). To confirm the functional impact of these deletions, we conducted dual luciferase reporter assays (DLRA) on a 1129-bp deletion in *Dna2*, a 2459-bp deletion in *Fan1*, and a 731-bp deletion in *Mcmdc*2. These deletions resulted in a significant reduction in luciferase activity, indicating a potential decrease in the activity of these genes (Fig. 3, C and F, and fig. S5C).

### Reduced intrachromatin interaction and fission

To explore the potential impact of chromatin interactions on chromosomal fission, we aligned the Hi-C reads of *M. psilurus* against the *M. aspalax* genome. The normalized contact maps for MaChr1 suggested two distinct interaction blocks with strong contacts within each block but weak interactions between the blocks (fig. S5D). In addition, we found significantly fewer intrachromosomal interactions within MaChr1 (between MaChr1a and MaChr1b; MaChr1a: Chr1: 1 to 160 Mb of *M. aspalax*, MaChr1b: Chr1: 160 to 193.8 Mb of *M. aspalax*) compared to interchromosomal interaction (between MaChr1a or MaChr1b and other nonfissioned chromosomes) (Fig. 3G).

### Fixation of the chromosomal fission

The broken terminal segment of MaChr1 remained intact and formed a small chromosome (MpChr32) in *M. psilurus* instead of being lost after the fission event. To understand why it was retained, we examined its gene composition and identified 253 functional genes on Chr32, which exhibited higher gene density (mean: 0.869 per 50-kb window) than the entire genome (mean: 0.846 per 50-kb window). Notably, MpChr32 harbored key genes including housekeeping gene actin beta (*Actb*) and the hypoxia adaptive gene Egl-9 family hypoxia inducible factor 1 (*Egln1*). To investigate how the chromosomal fission became fixed in *M. psilurus*, we investigated selective signals of *M. psilurus* for fissioned and nonfissioned chromosomes. Single-nucleotide polymorphisms (SNPs) with normalized cross-population number of segregating sites by length (XPNSL) value > 2 (see Materials and Methods) defined as positively selected sites in *M. psilurus* while normalized XPNSL value < −2 as positively selected sites in *M. aspalax*. The result suggested a higher density of selected SNPs in *M. psilurus* on MaChr1b region (homologous to MpChr32, 6.82 per 100-kb window) compared to other chromosomes (0.44 per 100-kb window) (Fig. 3H). In addition, we analyzed the selection timeline of all the *M. psilurus*–selective SNPs on the MaChr1b region and found that selection occurred from ~3.39 to 0.66 million years ago (fig. S5, E and F).

### Effects of fission on speciation

**Fig. 4.**
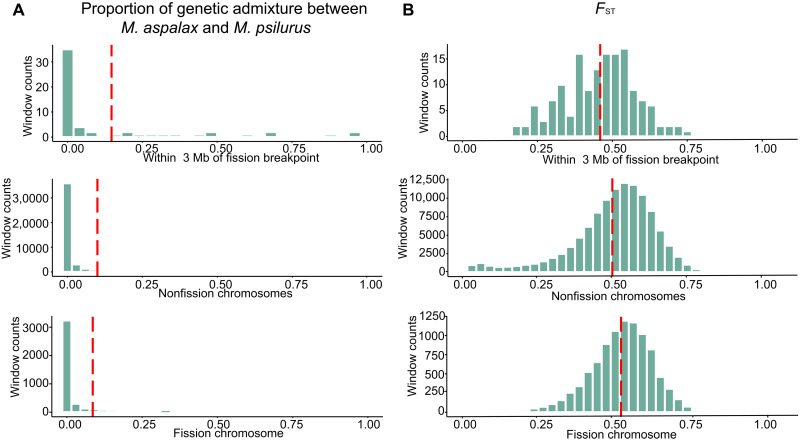
Effects of chromosomal fission on genetic admixture, recombination rate, and population differentiation. (**A**) Frequency distribution of genetic admixture proportion between *M. aspalax* and *M. psilurus* in regions within 3 Mb of the fission breakpoint on nonfissioned chromosomes and fission chromosome using a window size of 40 kb. No reduction in genetic admixture proportion is observed near the fission breakpoint but reduced on the entire chromosome. The *x* axis represents the range of genetic admixture proportions, while the *y* axis shows the number of windows, indicating the frequency of each admixture proportion range across 40-kb windows. The red dashed line marks the mean. (**B**) Distribution of *F*_ST_ between *M. aspalax* and *M. psilurus* in regions within 3 Mb of the fission breakpoint on nonfissioned chromosomes and fission chromosome with a window size of 40 kb. The *x* axis shows *F*_ST_ range, and the *y* axis indicates the number of windows. The red dashed line marks the mean.

To assess the impact of chromosomal fission on speciation, we examined population differentiation (*F*_ST_, *D*_xy_), genetic admixture, and recombination rate between two species in local genomic regions surrounding the fission breakpoint (± 1.5 Mb) and in nonfissioned chromosomes, respectively. Neither a reduction in genetic admixture (mean: 0.147 versus 0.100; Fig. 4A) nor a significant decrease in recombination rate (mean: 0.1419 versus 0.1426, Wilcoxon test: *P* value = 0.06709; fig. S5G) was detected around the fission breakpoint compared to nonfissioned chromosomes. Similarly, no increase in *D*_xy_ (mean: 0.391 versus 0.431; fig. S5H) or *F*_ST_ (mean: 0.459 versus 0.500; Fig. 4B) was observed near the fission breakpoint. However, when comparing the entire fissioned chromosome to all other chromosomes, we found significantly higher differentiation (*F*_ST_: mean: 0.529 versus 0.500, Wilcoxon test: *P* value < 2.2 × 10^−16^; Fig. 4B; *D*_xy_: mean: 0.446 versus 0.431, Wilcoxon test: *P* value < 2.2 × 10^−16^; fig. S5H), along with significantly lower genetic admixture (mean: 0.086 versus 0.100, Wilcoxon test: *P* value < 2.2 × 10^−16^; Fig. 4A) and recombination rates (mean: 0.1415 versus 0.1426, Wilcoxon test: *P* value < 2.2 × 10^−16^; fig. S5G).

### Demographic history contributing to population divergence

**Fig. 5.**
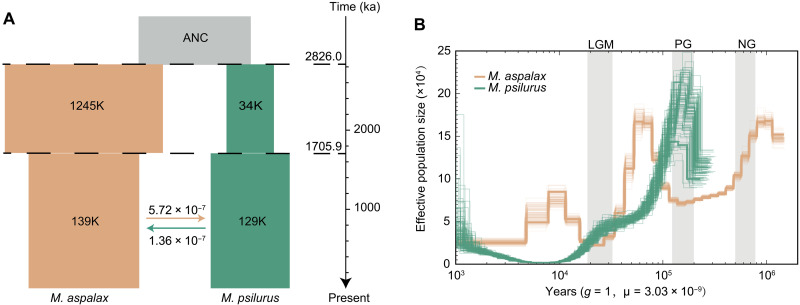
Population divergence and demographic history. (**A**) Population demographic simulation identified the secondary contact model as the best fit. This model simulates effective population size (indicated by the numbers in the bars), divergence time, secondary contact time (numbers above the dashed lines), and gene flow between the two species (numbers above the arrow symbols). Gene flow occurred only during the second phase. The bar on the right designates the timescale from the past to the present. (**B**) Fluctuations in effective population size for *M. aspalax* (in brown) and *M. psilurus* (in green). Gray shading indicates three glacial periods: the Last Glacial Maximum (LGM), the Penultimate Glacial (PG), and the Naynayxungla Glaciation (NG).

To assess the roles of geographic barriers and the fission event in the speciation of sister species, we examined the demographic histories of the fission-bearing *M. psilurus* and its sister species, *M. aspalax*. Our results indicate that they diverged ~2.8 million years ago [95% confidence interval (CI) at 2.37 to 4.59 million years ago] (Fig. 5A). No gene flow was detected during the early stages of divergence; however, recent gene flow has occurred between the two species (Fig. 5A) despite their karyotypic differences. This suggests that speciation began with geographic isolation, followed by secondary contact that facilitated gene flow. STRUCTURE analysis indicated individuals with minor genetic traces from the other species (fig. S5I), supporting the possibility of introgression, consistent with their overlapping habitats (Fig. 1A) and the limited gene flow observed (Fig. 5A). Although both species experienced reduced effective population sizes (*Ne*) during past glaciation events, they displayed different demographic trajectories (Fig. 5B), likely due to their contrasting distribution ranges (Fig. 1).

## DISCUSSION

Chromosomal fissions and fusions, although widespread across species, remain mechanistically enigmatic, with their evolutionary implications in speciation poorly resolved (*35*). While the mechanisms of chromosomal fusion have been studied extensively, those of chromosomal fission remain largely unexplored. Our study bridges this gap by identifying key genetic drivers of fission and its interplaying with geographic isolation in speciation dynamics of one zokor species.

### Genetic drivers of fission and neo-telomere formation

ITS likely acted as a fragile chromosomal breakage site (*14*). In *M. psilurus*, a single-nucleotide mutation disrupted an ESE in *Aplf*, generating an alternative splice variant that coexists with the wild-type transcript (*36*, *37*). This splicing defect introduced a frameshift mutation and PTC in exon 7, truncating the PBZ domain essential for recruiting DNA repair proteins to DSBs (*38*). Consequently, *Aplf* dysfunction impaired DSB repair (*17*), as confirmed by cellular assays (Fig. 2J), consistent with elevated *Aplf* expression in *M. psilurus* (Fig. 2, B and C)—a compensatory response to chronic DNA damage possibly due to a mixture of normal and abnormal transcripts. Although the truncated transcript leading to DNA repair failure has been validated at the cellular level, further confirmation of the entire process—incorporating both the normal and abnormal transcripts—through gene editing in mice could provide more definitive evidence.

Failed DSB repair can lead to the formation of neo-chromosomes, facilitated by the acquisition of functional neo-telomeres (*39*, *40*). Telomeric repeats can act as seed sequences inducing the formation of functional neo-telomeres at previously interstitial sites (*14*, *41*, *42*). In *M. psilurus*, the ITS not only predisposed the chromosome to breaks but also provided telomeric repeats that initiated neo-telomere formation (*43*). Notably, we detected a large intronic SV deletion in the gene *Dna2*, which reduced its activity in *M. psilurus*. *Dna2* is critical for homology-directed repair (*44*) and its SV likely amplifies this process, consistent with previous findings that *Dna2* knockdown increases neo-telomere formation (*14*). We also identified SVs in other genes likely involved in neo-telomere formation in *M. psilurus*, such as *Fan1* and *Mcmdc2*, which also affected their gene activity. *Fan1* has been reported to compensate for *Exo1* in telomere dynamics (*45*), while *Mcmdc2* plays a crucial role in meiotic DSB repair through homologous recombination (*46*). The precise mechanisms of these SVs in the neo-chromosome formation of *M. psilurus* require further investigation. In addition, if telomerase generates a functional telomere at a DSB, then the small chromosomal segment distal to the break is more likely to be lost (*14*). In the case of *M. psilurus*, the newly originated small chromosome-Mpchr32–harbored vital genes, and its loss may be lethal to animals. In addition, strong selection signals were found in Mpchr32 (Fig. 3H); this suggests that the fission is adaptive, potentially facilitating its spread and fixation (*20*).

### Chromosomal fission and speciation dynamics

Genomic studies on the effects of chromosomal number variations on gene flow have historically faced limitations due to challenges in obtaining high-quality chromosome-level genome assemblies in nonmodel systems, accurately identifying fusion and fission breakpoints, and lacking genomic evidence for gene flow between ancestral chromosomes and neo-chromosomes (*24*). In this study, the two high-quality chromosome-level genome assemblies of sister zokor species enabled precise identification of chromosomal number variations and breakpoints. The reconstruction of ancestral karyotypes and synteny analysis provided solid evidence for a fission event in *M. psilurus* compared with *M. aspalax*. Our assessment of the impact of chromosomal fission on speciation revealed no evidence of reduced recombination or genetic admixture near the fission breakpoint. However, we observed decreased genetic admixture and elevated genetic differentiation at the chromosomal level between the ancestral chromosome and the two neo-chromosomes when compared with other intact chromosomes. This suggests that chromosomal fissions and fusions may drive population divergence by inducing meiotic errors through aneuploidy and unsynapsed homologs, which can reduce hybrid fertility (*21*, *22*).

**Fig. 6.**
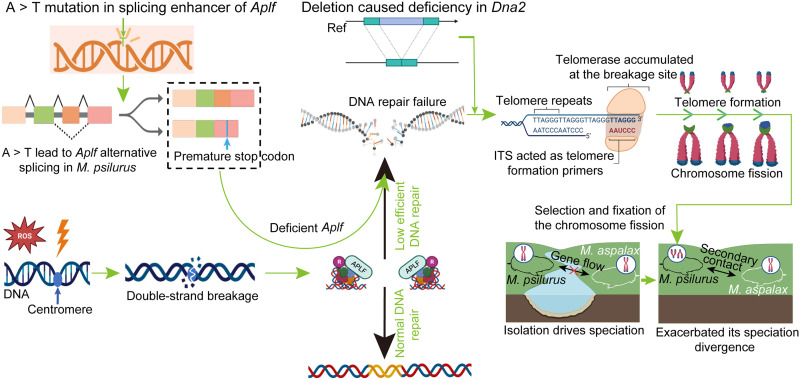
Schematic summary of our findings. A point mutation in the splicing enhancer of the *Aplf* gene induces alternative splicing, producing a truncated transcript with a premature stop codon. This truncation likely impairs DNA repair functionality. In addition, a deletion in *Dna2* reduces its activity, which may facilitate telomerase accumulation at chromosomal breakage sites. The presence of an ITS at the breakpoint serves as a primer for telomere formation, promoting neo-telomere establishment and, consequently, chromosomal fission. These two species experienced early geographic isolation and the fission event occurred during secondary contact.

Despite the karyotypic difference, complete reproductive isolation between the two zokor species has not occurred, as we detected recent gene flow between *M. psilurus* and *M. aspalax* when their distribution ranges overlapped. No gene flow was detected during the initial stages of divergence (Fig. 5A), indicating that geographic isolation likely accompanied this early divergence, while secondary contact in distributional ranges of the two species led to recent gene flow. Our results suggested that the chromosomal fission occurred after the initial divergence, which may have contributed to the later gene flow barriers. Thus, while early geographic isolation was a primary driver of divergence between these species, the fission event may have accelerated the latter process. In the absence of geographic isolation, chromosomal fissions could also become fixed because of the positive selection (Fig. 3 and fig. S5, E and F) and impede gene flow, similarly leading to speciation but over a longer timeframe. Overall, by integrating comparative genomics, functional assays, and population analyses, this study delineates a mechanism for chromosomal fission involving *Aplf* splicing defects and *Dna2* dysregulation in one zokor species (Fig. 6) and its potential role in speciation, which aligns with the sequence of events inferred from time estimates. Our findings highlight the synergistic roles of geographic isolation and chromosomal fission in speciation dynamics of zokors. Future studies should explore whether similar mechanisms underpin fission in diverse taxa, particularly in contexts of environmental stress and adaptive radiation.

## MATERIALS AND METHODS

### Sample collection and DNA sequencing

All procedures applied in sample collection and animal experiments were reviewed and approved by Institutional Ethics Committee of the College of Ecology, Lanzhou University (approval no. EAF2020003). A total of 27 individuals from two species were collected in China’s Inner Mongolia Autonomous Region and Hebei Province in 2020, which included 14 *M. psilurus* and 13 *M. aspalax* individuals. The collection sites were spaced at least 25 km apart between any two individuals. Latitude and longitude coordinates were recorded for each site, and 19 climate variables were retrieved for each site from WorldClim historical climate data (data S1). Live-captured animals were anesthetized using 2% α-chloralose and 10% urethane (8 ml/kg), after which tissue samples were collected and immediately preserved in liquid nitrogen. These flesh tissue samples were used for DNA extraction and next-generation resequencing across all the individuals (table S1). In addition, high–molecular weight DNA was extracted from flesh tissues of one male *M. aspalax* individual and one female *M. psilurus* individual for PacBio HiFi sequencing, enabling de novo genome assembly of each species. In parallel, long-read DNA sequencing was performed on flesh tissue samples from two *M. aspalax* individuals and three *M. psilurus* individuals to identify SVs (table S1). Principal components analysis of the 19 climatic variables across the species’ distribution areas was performed using the prcomp package in R (*47*).

### Estimation of genome size

To estimate the genome size, Jellyfish v2.2.0 (*48*) was used to generate a 21-nucleotide oligomer frequency distribution (-m 21) based on ~50× next-generation resequencing data (table S19) from the same individual used for the PacBio HiFi sequencing. Genome size and heterozygosity for the two species were then estimated using genome characteristics estimator (GCE) v1.0.2 (https://github.com/fanagislab/GCE) based on the K-mer frequency distribution.

### Draft genome assembly for the two species

The draft genomes of *M. aspalax* and *M. psilurus* were assembled using HiFi reads (tables S3 and S4). Raw sequencing data were obtained from the PacBio Sequel II platform and preprocessed with SMARTlink v9.0 (www.pacb.com/support/software-downloads) to obtain subread sequences using the following parameters: minimum subread length = 50, maximum subread length = 50,000, minimum number of passes = 3, and minimum predicted accuracy = 0.99. CCS v6.3.0 (https://github.com/PacificBiosciences/ccs) was then used to correct subreads sequences with default parameters, generating long (~12 kb) and highly accurate (>99%) HiFi reads. The HiFi reads in binary alignment/map (BAM) format were converted to FASTQ format using the BAM2fastx tool (https://github.com/PacificBiosciences/bam2fastx). HiFiasm v0.16.1 (*49*) was subsequently used to assemble the HiFi reads with default parameters. The completeness of the two draft genome assemblies was assessed using BUSCO (*50*) v4.1.4 with the mammalia_odb10 database. In addition, genome sizes, GC content, and N50 values for two draft genome assemblies were estimated with QUAST v5.2.0 (*51*).

### Chromosomal-level assembly for two species using Hi-C technology

Raw Hi-C reads were filtered using fastp v0.20.1 (*52*) to remove adapter sequences and low-quality reads (tables S7 and S8). Based on ~300 Gb of Hi-C reads, valid Hi-C pairs for *M. aspalax* and *M. psilurus* were obtained by aligning the reads to their respective draft genomes using Burrows-Wheeler Aligner (BWA) v0.7.17 (*53*). The Juicer v1.6 pipeline (*54*) was applied to identify valid chromatin interactions, which were subsequently used to anchor contigs to pseudo-chromosomes using the 3D DNA v18.9.22 pipeline (*55*) with default parameters. Last, contig misassemblies and scaffold misjoins were manually corrected with Juicebox v2.13.07 (*56*) based on chromatin interaction densities, resulting in the final high-quality chromosome-level assemblies.

### Repeat annotation

We used a combination of de novo and homology-based approaches to identify repetitive sequences in the *M. aspalax* and *M. psilurus* genomes. For de novo prediction, RepeatModeler v1.0.11 (*57*) was used to construct a repeat library for each genome. This was followed by RepeatMasker v4.0.7 (*58*), which searched for repeats using both the Repbase library (v20181026) and the self-trained repeat databases. In addition, RepeatProteinMask (a tool within RepeatMasker package) was applied to annotate repeats based on the TE protein database. Tandem repeats were further annotated using Tandem Repeat Finder v4.0.9 (*59*). After validating the genome coordinates, we generated a final nonredundant annotation of the repetitive sequences.

### LTR annotation

LTR-RTs were first identified using LTR_FINDER v1.06 (*60*). To enhance the prediction of LTR-RTs in each genome, LTRharvest v1.5.10 (*61*) was subsequently used. Last, LTR_retriever v1.9 (*62*) was used to integrate the results from LTR_FINDER and LTRharvest, filtering out false positives to ensure accurate LTR-RT annotations.

### Gene prediction and annotation

We used a combination of transcriptome-based, homology-based, and ab initio approaches to predict protein-coding genes in the *M. aspalax* and *M. psilurus* genomes. For transcriptome-based prediction, HISAT v2.0.4 (*63*) and StringTie v1.2.3 (*64*) were used for reference-guided RNA transcript assembly. Candidate protein-coding regions within the assembled transcripts were identified using TransDecoder v2.0 (https://github.com/TransDecoder/TransDecoder). In addition, de novo assembled transcripts generated by Trinity v2.5.1 (*65*) were aligned to the genome assemblies for gene prediction using PASA v2.0.2 (*66*).

For ab initio prediction, BUSCO v4.1.4 (*50*) was initially used to train a species-specific gene prediction model for each species based on the mammalia_odb10 database. Following this, AUGUSTUS v2.5.5 (*67*) was used to predict genes using both the species-trained model and the human model from the database, ensuring comprehensive gene prediction.

For homology-based gene prediction, genome sequences and annotations from seven species—*Mus Musculus* (GCA_000001635.9), *Rattus rattus* (GCA_011064425.1), *Mesocricetus auratus* (GCF_017639785.1), *Cricetulus griseus* (GCF_000223135.1), *Spalax galili* (GCA_000622305.1), *Rhizomys pruinosus* (GCA_009823505.1) and *E. fontanierii* (GCA_035773235.1)—were downloaded from National Center for Biotechnology Information (NCBI). GeMoMa v1.3.1 (*68*) was applied to infer protein-coding genes based on these related species. In addition, Exonerate v2.4.0 (www.animalgenome.org/bioinfo/resources/manuals/exonerate) was used to align the assembled genomes with high-quality homologous proteins from the UniProt database (www.uniprot.org), aiding in gene prediction.

All evidence from transcriptome-based, homology-based, and ab initio approaches were integrated using EVidenceMolder v1.1.1 (*66*) pipeline to generate a final consensus gene set. Last, untranslated region annotations and spliced isoforms were incorporated using PASA v2.0.2 (https://github.com/PASApipeline/PASApipeline). Gene symbols for the annotated genes were assigned by aligning the predicted proteins with the SwissProt database (www.uniprot.org).

### Constructing phylogenetic trees using cytochrome *b* gene

We performed next-generation sequencing on *M. myospalax* flesh tissue (table S20). After assembling and annotating mitochondrial genomes using MitoHiFi v3.2.1 (https://github.com/marcelauliano/MitoHiFi), we extracted cytochrome *b* gene sequences from *M. aspalax*, *M. psilurus*, and other six *Eospalax* species using gffread (http://ccb.jhu.edu/software/stringtie/gff.shtml#gffread). The cytochrome *b* gene sequences of *R. sumatrensis* (GenBank accession number: NC_039104.1, nucleotide position: 14,148 to 15,287) obtained from the NCBI database were used as the outgroup for phylogenetic analysis. We aligned the clean reads [filtered by fastp v0.20.1 (*52*)] to the cytochrome *b* gene sequence of *M. aspalax* using BWA v0.7.17 (*53*). The cytochrome *b* consensus sequence for *M. myospalax* was generated from the BAM file using ANGSD v0.940 (*69*). Last, the cytochrome *b* gene sequences of the 10 species were input into MEGA v11 (*70*) to construct a neighbor-joining (NJ) tree.

### Genome collinearity

Using the *M. aspalax* genome as a reference, we performed pairwise alignments with the *M. psilurus* genome and six other *Eospalax* species (*E. baileyi*, *E. smithi*, *E. cansus*, *E. rufescens*, *E. rothschildii*, and *E. fontanierii*). The alignments were generated using Mummer v4.0.0 (*71*) with default parameters, and those shorter than 8 kb were filtered out. JCVI v1.2.7 (*72*) was then used to visualize the collinear blocks through dot plots and synteny plots. In addition, based on the dot plots, we adjusted the positive and negative strands, using *M. aspalax* as the reference, to improve the synteny plot visualization.

### WGDI analysis and ancestral karyotype reconstruction

*E. fontanierii*, *E*. *cansus*, *Spalax carmeli*, and *R. norvegicus* were selected as outgroup species. After obtaining the longest transcripts of each gene for the four outgroup species and the two study species, Diamond v2.0.13 (*73*) was used to align *M. aspalax* and *M. psilurus* with the four outgroups respectively. Subsequently, WGDI v0.6.1 (*33*) was used to visualize gene collinear blocks using dot plots. Based on these dot plots, we inferred the ancestral karyotype of the common ancestor of *M. aspalax* and *M. psilurus*.

### Identification of telomeric repeat sequences

Based on the common vertebrate telomeric repeat unit (TTAGGG)*n*, tidk v0.2.1 (*74*) was applied to identify telomeric repeat sequences in *M. aspalax*, *M. psilurus*, and six other genomes within the *Eospalax* genus.

### Genomic localization of centromeres

We performed ChIP-seq using the centromere protein-A (CNEP-A) antibody (Cell Signaling Technology, #2047) on frozen liver tissues (−80°C) from *M. aspalax* and *M. psilurus*. After filtering with fastp v0.20.1 (*52*) (-5 --cut_front_window_size 4 --cut_front_mean_quality 20–3 --cut_tail_window_size 4 --cut_tail_mean_quality 20 --cut_right --cut_right_window_size 4 --cut_right_mean_quality 20 --detect_adapter_for_pe -q 15 -u 40 -e 20 -n 5 -l 30 -p -P 20 -w 20), we obtained 25,729,787 and 18,960,924 high-quality reads for *M. aspalax* and *M. psilurus*, respectively. The reads were aligned to their respective reference genomes using Bowtie2 v2.4.4 (*75*), and sequence alignment/map (SAM) files were converted to sorted BAM files using SAMtools v1.6 (*76*). Histone mark signals were quantified using bamCoverage in DeepTools v3.5.1 (*77*) (--binsize 20000 --normalizeUsing RPKM).

### Different expression analysis

Bulk RNA-seq was performed on flesh, liver, lung, and heart tissues at −80°C, with at least two biological replicates for both *M. aspalax* and *M. psilurus*, using the DNBSEQ-T7 platform (table S16). Raw sequencing reads were filtered to remove adapters and low-quality sequences using fastp v0.20.1 (*52*). The clean reads were then mapped to the reference genome of *M. aspalax* and *M. psilurus*, respectively, using HISAT v2.2.1 (*63*) with default parameters. StringTie v2.2.1 (*64*) was used to calculate and quantify the expression levels of all transcripts in each tissue type using fragments per kilobase of transcript per million mapped reads (FPKM) with default parameters.

### Splicing isoform detection

Nanopore long-read RNA sequencing (Iso-seq), generating 6 Gb of data, was conducted on frozen flesh tissue (−80°C) from individual *M. aspalax* and *M. psilurus* using the Nanopore PromethION platform (table S17). Raw sequencing reads were quality-filtered using Trimmomatic (*78*). For transcriptome assembly, we used a reference-guided approach using wf-transcriptomes pipeline v1.1.1 (https://github.com/epi2me-labs/wf-transcriptomes). Transcripts were annotated by assigning labels and corresponding gene identifiers based on the reference genome annotation provided in this study.

### Identification of gene loss

To generate pairwise alignment chains as input for Tool To Infer Orthologs From Genome Alignments (TOGA; v1.1.1) (*79*), we initially used the make_chains.py script (*79*) to align the assembled full-length transcripts of *M. aspalax* and *M. psilurus* against the mouse soft-masked genome (mm10, available at https://hgdownload.cse.ucsc.edu/goldenpath/mm10/bigZips/mm10.2bit). Subsequently, we applied TOGA (*80*) to identify lost genes in *M. aspalax* and *M. psilurus* using mm10.wgEncodeGencodeCompVM25.bed as the input BED12 file for mouse annotation and mm10.wgEncodeGencodeCompVM25.isoforms.txt as the input isoform file for mouse, both provided by the TOGA repository. Last, we filtered and selected completely lost genes for further downstream analysis.

### Identification of SVs

#### Identification of interspecific SVs based on long-read DNA sequencing data

Long-read data from two *M. aspalax* individuals, three *M. psilurus individuals* (table S18), and 17 previously published long-read sequencing data from six *Eospalax* species were aligned to the *M. aspalax* reference genome using NGMLR v0.2.7 (*81*), and SVs for each individual were detected using Sniffles v1.0.12 (*81*). SURVIVOR v1.0.7 (*82*) was then applied to merge SVs from all individuals, generating a consensus set of SVs. Sniffles v1.0.12 was subsequently rerun to identify individual-specific SVs and Implement for Refining Insertion Sequences (IRIS v1.0.4) (https://github.com/mkirsche/Iris) was used to correct insertions and deletions.

#### Identification of interspecific SVs based on short-read DNA sequencing data

We also used short-read data from 27 individuals of two species (table S19) to identify interspecies SVs. The short-read data were first aligned to the *M. aspalax* reference genome using Burrows-Wheeler Aligner - Maximal Exact Match (BWA-MEM) v2.21 (*53*), and the resulting BAM files were sorted using SAMtools v1.6 (*76*), with PCR duplicates removed using GATK v4.1.4.1 (*83*). SVs were identified using DELLY2 v1.0.3 (*84*), LUMPY v0.2.13 (*85*) and MANTA v1.0.1 (*86*). The SVs identified by all three programs were integrated using SVIMMER v0.1 (https://github.com/DecodeGenetics/svimmer). GRAPHTYPER v2.7.3 (*87*) was then used to genotype the integrated SVs. A series of filtering steps were applied to create a high-quality short-read SV dataset: (i) filtering out SVs with a missing rate greater than 0.05 and (ii) removing SVs shorter than 50 bp. This analysis resulted in the identification of 115,366 SVs from the short-read data. In addition, we repeated the same pipeline after incorporating 146 additional short-read datasets from the *Eospalax* genus, which were obtained from published studies (*30*, *88*) These datasets included 55 *E. baileyi*, 25 *E. cansus*, 21 *E. smithi*, 18 *E. rothschildi*, 14 *E. rufescens*, and 13 *E. fontanierii* individuals. SVs were identified using *M. aspalax* as the reference genome.

#### Annotating SVs

The identified SVs were annotated using the VCFANNO v0.3.3 (*89*). Gene regions, exon regions, coding sequences (CDSs) start sites, CDS stop sites, regions 2-kb upstream and downstream of genes, and intergenic regions were first extracted based on the reference genome annotation. The SVs were then mapped to these regions using VCFANNO v0.3.3 (*89*) to provide detailed functional annotations.

#### Screening of candidate SVs

A frequency-based approach was used to screen candidate SVs from both long-read and short-read datasets by calculating the occurrence frequency of each SV within each species using custom in-house scripts. We focused on identifying SVs that were fixed in *M. psilurus* but completely absent in the other seven species. Functional enrichment analysis for genes closest to these fixed SVs was then conducted using Metascape (https://metascape.org/gp).

### Hi-C data analysis

#### Construction of normalized interaction matrices

HiC-Pro v3.1.0 (*90*) was used to construct contact matrices based on the high-quality chromosome-level genomes and Hi-C data. Bowtie2 v2.4.4 (*75*) was first used to align the Hi-C data to the genome. After removing unmatched read pairs, low-quality read pairs, read pairs with multiple alignments, and singleton read pairs, we retained uniquely aligned read pairs to match Mbo I restriction enzyme. In addition, three invalid pair types (dangling end, self circle, and dumped pairs) were filtered out, resulting in 409,605,081 and 493,301,206 valid pairs, respectively, for constructing raw interaction matrices at various resolutions (1 Mb, 100 kb, 40 kb, 20 kb, and 10 kb). Raw contact matrices were then normalized using iterative correction and eigenvector decomposition (ICE) methods.

#### Comparison of significant interactions

FitHic v2.0.0 (*91*) was used to calculate significant interactions from 20-kb resolution interactions matrices, with a significance threshold of *P* < 0.01 and a false discovery rate < 0.01. Significant interactions were required to be supported by at least five valid pairs.

### Identification of SNPs

We used BWA-MEM v2.21 (*53*) to map filtered short-read DNA sequencing reads for each individual (table S19) to the *M. aspalax* reference genome with default parameters. SAMtools v1.6 (*76*) was used to sort the mapped reads, and MarkDuplicates module in GATK v4.1.4.1 (*83*) was used to remove PCR duplicates. Subsequently, SNPs were identified for each individual using GATK HaplotypeCaller. To reduce false positives, several filtering steps were applied, including the removal of: (i) indels with a quality score < 3, (ii) SNPs with more than two alleles, (iii) SNPs located within 5 bp of any indels, (iv) SNPs with a genotyping quality score (GQ) < 10, and (v) SNPs with extremely low (<^1^/_3_ average depth) or extremely high (>threefold average depth) coverage. Afterward, GATK’s GenotypeGVCFs tool was used to merge individual results and identify multisample SNPs using both modes: (i) with the -all-sites parameter to output all genomic sites and (ii) without the parameter to exclusively output SNP sites. These multisample SNPs were filtered using the GATK VariantFiltration module with the following parameters: “QD < 2.0 || FS > 200.0 || ReadPosRankSum < −20.0 || QD < 2.0 || FS > 60.0 || MQ < 40.0 || MQRankSum < −12.5 || ReadPosRankSum < −8.0”. Last, soft filtering was performed using VCFtools v0.1.16 (*92*) with the parameters “--maf 0.05, --minDP 5, --maxDP 150, --minGQ 20, --hwe 0.001 --max-missing 0.95”.

### Analysis of genetic diversity and population differentiation

We assessed genetic differentiation between the two populations using *F*_ST_ and *D*_xy_. *F*_ST_ was calculated using VCFtools v0.1.16 (*92*), while *D*_xy_ was estimated using the Python script *popgenWindows.py* from the *genomics_general* package (https://github.com/simonhmartin/genomics_general). For recombination rate analysis, the VCF file was phased using Beagle v4.0 (https://faculty.washington.edu/browning/beagle/beagle.html), and recombination rate parameters were calculated with ReLERNN v1.0.0 (https://github.com/kr-colab/ReLERNN). In addition, RFmix (*93*) was applied to detect signals of genetic admixture between *M. psilurus* and *M. aspalax*. All these parameters were calculated for the local genomic regions surrounding the fission breakpoint (± 1.5 Mb), nonfissioned chromosomes, and fissioned chromosomes.

### Demographic history

For the two species analyzed in this study, paired-end short-read DNA sequencing reads with the highest coverage (table S19) were selected for demographic analysis using the pairwise sequentially Markovian coalescent (PSMC) (*94*) model (v0.6.5). Initially, the mpileup module in SAMtools v1.6 (*76*) was used to convert the consensus sequences from BAM format to binary variant call format (BCF) format, followed by conversion to VCF format using BCFtools v1.10 (*95*). The script vcfutils.pl was then used to transform the consensus sequences into a fastq-like format, with a minimum read depth (-*d*) set to one-third of the average depth, and a maximum read depth (-*D*) to twice the average depth. Next, the fq2psmcfa and splitfa modules in the PSMC package were used to generate input files required for the PSMC simulation. We ran the PSMC model 100 times to enable random sampling of short fragments with the following command: “for i in 1..100; do psmc -N25 -t15 -r5 -b -p “4+25*2+4+6” -o $i.psmc split.psmcfa; done.” Last, the output results from all runs were concatenated, and the psmc_plot.pl script from the PSMC package was used for visualization, with the mutation rate (μ) setting to 3.03 × 10^−09^ and the generation time to 1 year (*30*). In addition, we evaluated the influence of climate events on the effective population size of each species by incorporating three glacial periods: Naynayxungla [780 to 500 thousand years (ka)], Penultimate (195 to 135 ka) (*96*), and Last Glacial Maximum (26.5 to 19.0 ka) (*97*).

We further estimated the population evolutionary history of *M. aspalax* and *M. psilurus* using the ∂a∂i pipeline (v3.1.6) (*98*). The input file was generated for both species from the final SNP datasets (VCF file) using vcf_to_dadi.py script (https://ppp.readthedocs.io/en/latest/PPP_pages/Input_File_Generators/vcf_to_dadi.html). We calculated the likelihood of the observed SFS under 20 different candidate demographic models (fig. S6), running 100 independent simulations from different starting points for each model. The best-fitting demographic model was identified on the basis of the lowest Akaike information criterion score (data S2).

### Estimation of the fission time

Using the *M. aspalax* soft-masked genome as a reference, we aligned the soft-masked genomes of *M. psilurus* and *E. fontanierii* (outgroup) to the reference using the make_chains.py script (*79*). Next, the doRecipBest.pl script (https://github.com/ucscGenomeBrowser/kent/blob/master/src/hg/utils/automation/doRecipBest.pl) was used to extract reciprocal best alignments from the chain file. The ChainToAxt command in the UCSC tools package (*99*) was then used to convert the chain file into an axt file. Subsequently, we applied the Tytus pipeline (*34*) to calculate the values of UBCS.

Specifically, the Tytus pipeline (*34*) was designed to first identify single-nucleotide differences (SNDs) using the reciprocal best alignments between *M. aspalax* and *M. psilurus*. The reciprocal best chain file was used to map *M. aspalax* genome regions to their homologs in the *E. fontanierii* genome. The SNDs were then filtered and classified as derived in either the target (*M. aspalax* genome) or the query (*M. psilurus* genome) based on the criteria suggested by the pipeline. Last, the UBCS statistics were calculated as the difference between the expected and observed number of bias-clustered substitutions (BCSs) in each 1-Mb window. In addition, BCSs were defined as those occurring within a 300-bp window containing at least four substitutions, with at least 80% of them being AT-to-GC substitutions. Based on calculated UBCS values, we used the following formula to estimate the fission time: $T=T_{\text{DIV}}\times \left(\;1-\frac{R1}{R2}\;\right)$ . In this calculation, *T*_DIV_ represents the divergence time between *M. aspalax* and *M. psilurus*. The parameter R1 corresponds to the ratio of UBCS values within 2-Mb region flanking the fission breakpoint for SNDs derived in *M. psilurus* relative to that derived in *M. aspalax*. The parameter R2 denotes the ratio of UBCS values for SNDs derived in *M. psilurus* between two specific regions: (i) the initial telomeric region and (ii) a corresponding region of identical length located several megabases away from the telomere (*34*).

### Time estimation of the site mutation of Aplf and selected signals on the MaChr1b segment

We estimated the divergence time for selected SNPs in *M. psilurus* by calculating the statistic *D*_a_, which represents the number of mutations accumulated since the two species diverged from their most recent common ancestor (*100*). The formula used was $D_{a}=D_{\mathrm{xy}}-\frac{\pi \left(x\right)+\pi \left(y\right)}{2}$ , where *D_xy_* is the divergence between the two species and π(*x*) and π(*y*) are the nucleotide diversities for each species. We calculated *D_xy_* for all sites using a 20-kb window with a 20-kb step. The divergence time was then estimated in generations using the formula $T=\frac{\mathrm{Da}}{2\mu }$ (*101*), where μ is the mutation rate (3.03 × 10^−09^ mutations per site per generation). The generation time was assumed to be 1 year per generation.

### Dual-Luciferase reporter assay

The reduced regulatory activity caused by the deletion of the *Dna2*, *Fan1*, and *Mcmdc2* genes was validated using a Dual-Luciferase Reporter Assay System. Reporter plasmids for the *Dna2*-nondel and *Dna2*-del were constructed by cloning the amplified *Dna2*-nondel sequence (1129-bp deletion with 400-bp flanking sequence) and *Dna2*-del sequence (400-bp flanking sequence only) into pGL3-promoter plasmids. Similarly, reporter plasmids for the *Fan1* gene deletion (2459 bp) and the *Mcmdc2* gene deletion (731 bp) were constructed. The pGL3-basic and pGL3-enhancer plasmids were used as negative and positive controls, respectively. For both the experimental and control groups, the pRL-TK plasmid was used as an internal reference to normalize firefly luciferase activity by dividing it by Renilla luciferase activity. HEK293T cells were cultured with Dulbecco’s Modified Eagle’s Medium (DMEM) medium (G4524-500ML) supplemented with 10% fetal bovine serum (AB-FBS-1050S) and 1% penicillin-streptomycin solution (C0222). Then, HEK293T cells were transfected using transfection using polyJet reagent (SL100688), and fluorescence values were measured 48 hours posttransfection using the Promega GloMax 20/20 Luminometer. Each group included at least three biological replicates.

### Experimental validation of isoforms

Flesh tissues from two *M. aspalax* individuals and three *M. psilurus* individuals were ground thoroughly with liquid nitrogen to ensure complete disruption, followed by sonication for 3 min. Total RNA was then extracted using the MolPure Cell/Tissue Total RNA Kit (19211ES) and reverse-transcribed into cDNA using the Hifair V one-step RT-gDNA digestion SuperMix for qPCR kit (11142ES10). RT-PCR was conducted with a forward primer targeting exon 5 and a reverse primer complementary to exon 7 of the *Aplf gene.* β-Actin was used as an internal control. The primer sequences are provided in table S21.

### Exon skipping experiments

#### In silico splicing assay

Putative ESEs for the DNA sequence of exon 6 of the *Aplf* gene in the two studied species were identified using ESEfinder (https://esefinder.ahc.umn.edu/cgi-bin/tools/ESE3/esefinder.cgi) with the serine- and arginine-rich (SR) protein matrices. Mutations that significantly reduced the ESE score were selected for further in vitro assays.

#### Plasmid construction

The sequences of target exon 6 of *Aplf* gene, either with or without the corresponding site mutations, along with 200-bp nucleotides of flanking shortened introns, were synthesized by Tsingke Biotech. Xho I (CCGCˆTCGAG) and Nhe I (CTAGˆCTAGC) restriction sites were added. The shortened introns were designed by NatGene (https://services.healthtech.dtu.dk/services/NetGene2-2.42) to avoid activation of cryptic splicing. The sequences were then cloned into the pSPL3 plasmid between exon A and exon B. The pSPL3 plasmid (P30680) was from MiaoLing Biology.

#### In vitro splicing assay

HEK293T and Hela cells were cultured with DMEM medium (G4524-500ML) supplemented with 10% fetal bovine serum (AB-FBS-1050S) and 1% penicillin-streptomycin solution (C0222). Once the cells reached 70% confluence in six-well plates, 2 μg of plasmids—either with or without the site mutations-were transfected into the HEK293T and HeLa cells using 4 μl of polyJet transfection reagent (SL100688). The empty pSPL3 plasmid vector was used as a positive control. Thirty-six hours after transfection, the total RNA was extracted and subsequently reverse-transcribed into cDNA. PCR was subsequently performed using a forward primer (TCTGAGTCACCTGGACAACC) targeting upstream exon A and a reverse primer (ATCTCAGTGGTATTTGTGAGC) targeting downstream exon B (*102*).

### CRISPR-Cas9 editing for *Aplf* gene

#### Plasmid construction

To investigate whether truncated *Aplf* gene in *M. psilurus* leads to functional deficiency, we generated an *Aplf* knockout model in mouse embryonic fibroblasts (3T3) using CRISPR-Cas9 technology. First, sgRNAs targeting the *Aplf* gene in the mm10 genome were designed using the CHOPCHOP web tool (https://chopchop.cbu.uib.no/). To create a knockout model that mimics the *Aplf* gene truncation observed in *M. psilurus*, six sgRNAs were selected to target regions near the position of the premature translation termination in exon 7. A mismatch tolerance of MM1 (mismatch = 1) was applied with a cutoff of 0. All sgRNAs were designed to end with the NGG sequence, which serves as the protospacer adjacent motif recognized by the Cas9 protein.

To maximize U6 promoter activity, we added CACCG to the 5′ end of the sense guide oligo and added AAAC to the 3′ end of antisense guide oligo (table S22). The sense and antisense guide oligos were synthesized by Tsingke Biotech, annealed to form double-stranded sgRNAs using Nuclease-Free Buffer (NFB) buffer, and cloned into the pX459V2.0 plasmid, which contains a puromycin-resistance gene for selection.

The constructed plasmids were transformed into DH5ɑ competent cells, followed by the addition of 1-ml LB medium for amplification with shaking for 30 min. The remaining 30 μl of the transformation precipitate obtained after centrifugation was plated onto solid LB medium and cultured overnight. Monoclonal bacterial colonies were then picked for PCR amplication, and positive clones were verified through sequencing by Tsingke Biotech. The forward primer used was TTCTCTGTGTAACCTAGGGTGCCCAGGAAC, and the reverse primer was GGTATTTAAGTCTGGATTCCCCAGTATTTG. Successfully constructed plasmids were amplified in 20-ml cultures of *Escherichia coli* and extracted using a high-purity plasmid small extract medium dose kit (DP107).

#### Cells transfection and gene knockout

Mouse embryonic fibroblasts (3T3) were obtained from the Cheng Bo Laboratory and cultured in DMEM medium (G4524-500ML) supplemented with 10% bovine serum (AB-FBS-1050S) and 1% penicillin-streptomycin solution (C0222). The correctly constructed plasmids, along with an empty pX459V2.0 plasmid, were transfected into 3T3 cells using polyJet transfection reagent (SL100688). Eight hours after transfection, the medium was replaced with fresh culture medium. Successfully transfected cells were selected using puromycin, with wild-type 3T3 cells serving as a negative control. A portion of the surviving cells was collected for genomic DNA extraction. PCR and Sanger sequencing were performed to verify whether the sgRNAs had correctly targeted and cut the genome at the intended sites. PCR amplification and Sanger sequencing were performed to verify whether the sgRNAs had correctly targeted and cut the genome at the intended sites. The forward primer used was TTCTCTGTGTAACCTAGGGTGCCCAGGAAC, and the reverse primer was GGTATTTAAGTCTGGATTCCCCAGTATTTG. The presence of missense mutations was indicated by spurious peaks in the sequencing chromatograms. Subsequently, the cells were diluted and plated at densities of 1000 and 3000 cells per 10-cm culture dish. Monoclonal cell colonies were then isolated, and genomic DNA was extracted from each colony. PCR amplification and Sanger sequencing were again performed to verify whether the sgRNAs had accurately targeted the genomic sites and introduced the desired mutations. The presence of single peaks in the sequencing results confirmed successful editing.

#### DNA damage–induced assay

Cells with successful *Aplf* gene knockout, along with control cells, were treated with 10 μM camptothecin for 0, 30, 60, and 90 min. After treatment, cells were harvested and lysed using radioimmunoprecipitation assay buffer supplemented with protease inhibitors to extract proteins. Following the addition of loading buffer, the samples were sonicated for 1 min to shear long DNA fragments and then boiled at 100°C for 10 min to denature the proteins.

Western blot was subsequently performed to detect the 𝛾H2AX signal using a primary antibody (MB0175) from Biogot Biotechnology and a secondary antibody from the Cheng Bo Laboratory. β-Actin was used as a loading control, with the primary antibody (66009*1) and secondary antibody (SA00001-1), both from Proteintech.
