# Supplementary material for: Aplf/Dna2 variants drive chromosomal fission and accelerate speciation in zokors
Source: Sci Adv. 2025 Sep 5;11(36):eadt2282. doi: 10.1126/sciadv.adt2282 (PMC12412657; doi:10.1126/sciadv.adt2282)
Supplement: Supplementary file 1 — Figs. S1 to S6 Tables S1 to S22 Legends for data S1 and S2 [file sciadv.adt2282_sm.pdf]

Science Advances
AAAS

Supplementary Materials for

Aplf/Dna2 variants drive chromosomal fission and accelerate speciation in zokors

Na Wan *et al.*

Corresponding author: Kexin Li, likexin@lzu.edu.cn; Jianquan Liu, liujq@nwipb.ac.cn

Sci. Adv. **11**, eadt2282 (2025)
DOI: 10.1126/sciadv.adt2282

The PDF file includes:

Figs. S1 to S6
Tables S1 to S22
Legends for data S1 and S2

Other Supplementary Material for this manuscript includes the following:

Data S1 and S2

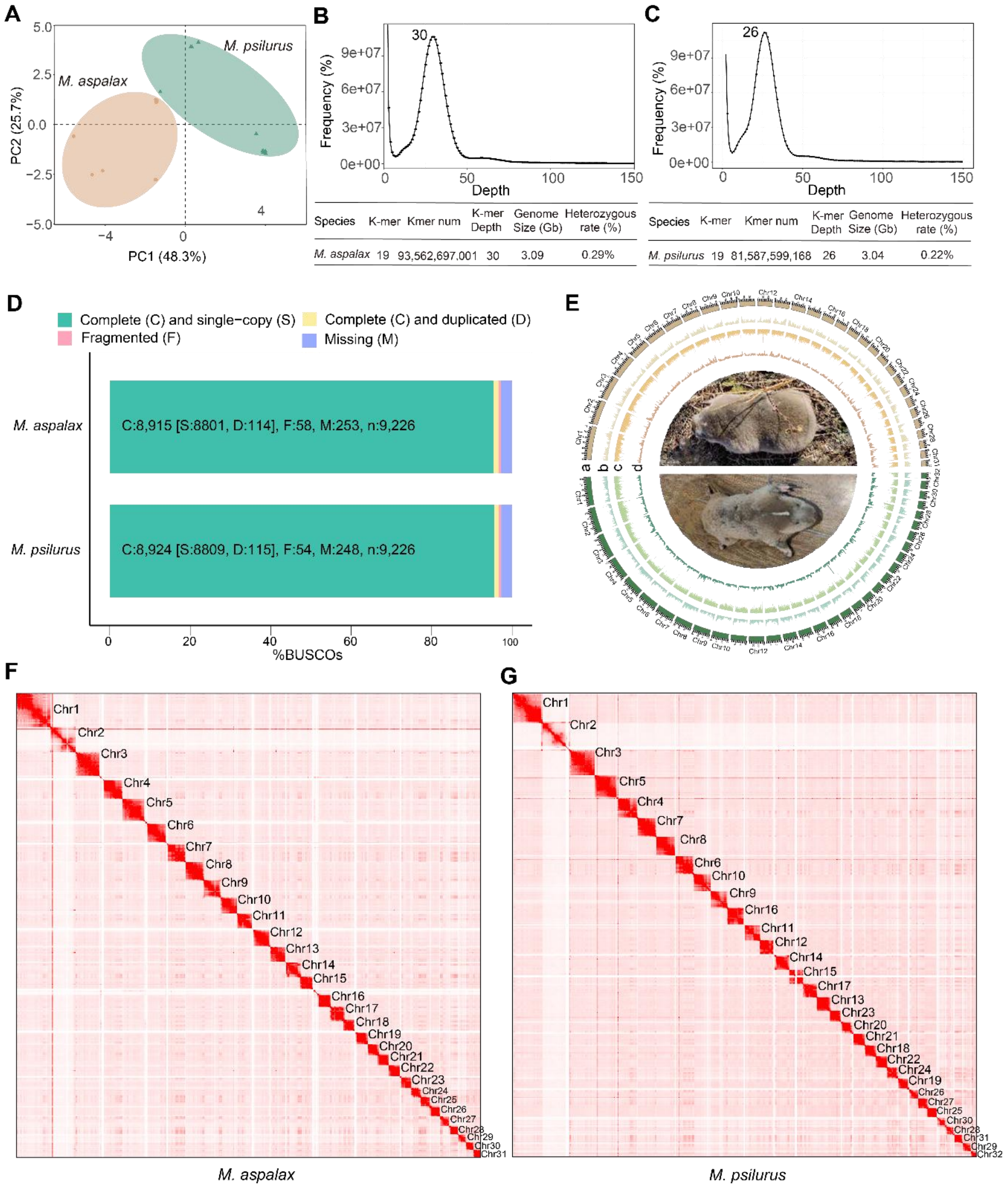

fig. S1.

Reference genome assembly of *M. aspalax* and *M. psilurus*. (A) Principal component analysis based on 19 climatic variables for *M. aspalax* and *M. psilurus*, illustrating environmental differentiation between the two species. **(B)** Genome size estimation of *M. aspalax* based on K-mer distribution. **(C)** Genome size estimation of *M. psilursus* based on K-mer distribution. **(D)** BUSCO assessment of *M. aspalax* and *M. psilurus* assemblies. **(E)**

Circos plot illustrating the genomic features of the two study species, with *M. aspalax* represented at the top (in brown) and *M. psilurus* at the bottom (in green). From outer to inner rings: (a) Chromosome sizes of 31 chromosomes in *M. aspalax* and 32 chromosomes in *M. psilurus*; (b) GC content; (c) distribution of repetitive elements; (d) gene density. **(F)** Hi-C interaction heatmap of the *M. aspalax* genome, illustrating chromatin contact frequencies across the 31 chromosomes. **(G)** Hi-C interaction heatmap of the *M. psilurus* genome, showing chromatin interaction patterns across the 32 chromosomes.

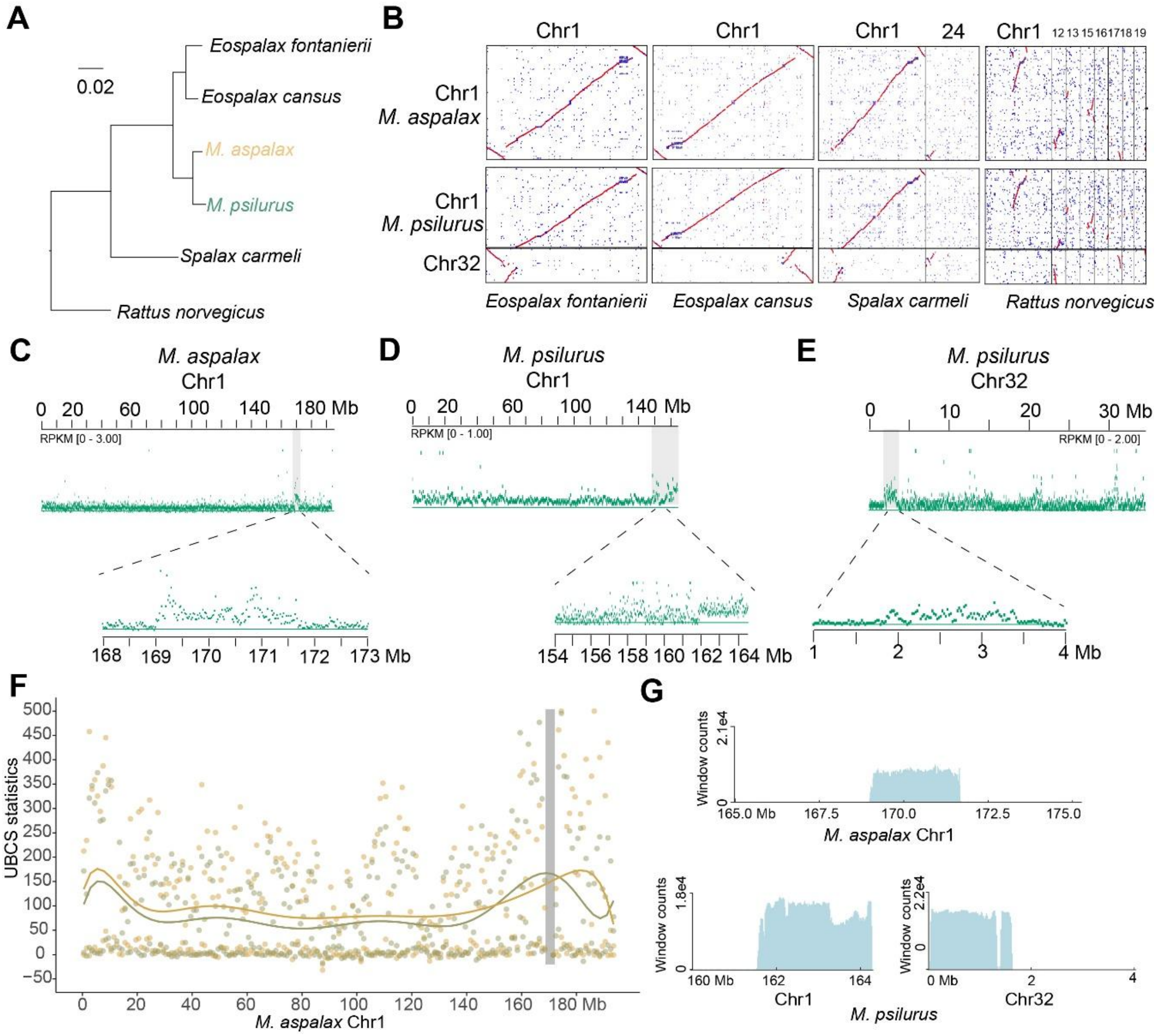

fig. S2.

Validation for the chromosome fission event and centromere identification. (A) Phylogenetic tree constructed based on genome assemblies from four outgroups (*Eospalax fontanierii*, *Eospalax cansus*, *Spalax carmeli*, *Rattus norvegicus*) and two study species (*M. aspalax* and *M. psilurus*). **(B)** The collinear dot plot between four outgroups and *M. aspalax*, *M. psilurus* respectively provided evidence for the fission event. **(C)** ChIP-Seq analysis reveals a peak enrichment near the fission breakpoint on chromosome 1 of *M. aspalax* (MpChr1: ~169 Mb – 171.5 Mb). **(D)** ChIP-Seq analysis identifies a peak enrichment near the terminal region of chromosome 1 in *M. psilurus* (MpChr1). **(E)** ChIP-Seq analysis detects a peak enrichment near the terminal region of chromosome 32 in *M. psilurus* (MpChr32). **(F)** Values of Unexpected Bias Clustered Substitutions (UBCS) for substitutions derived in *M. aspalax* (yellow) and *M. psilurus* (green) across the entire *M. aspalax* chromosome 1. Dots represent UBCS values, and lines indicate UBCS statistics

smoothed using loess regression. The grey shaded region highlights the fission breakpoint (MaChr1: 169Mb - 172Mb). **(G)** Predicted telomeric sequences enriched at the MaChr1 breakpoint (top) and chromosome ends of MpChr1 and MpChr32 (bottom).

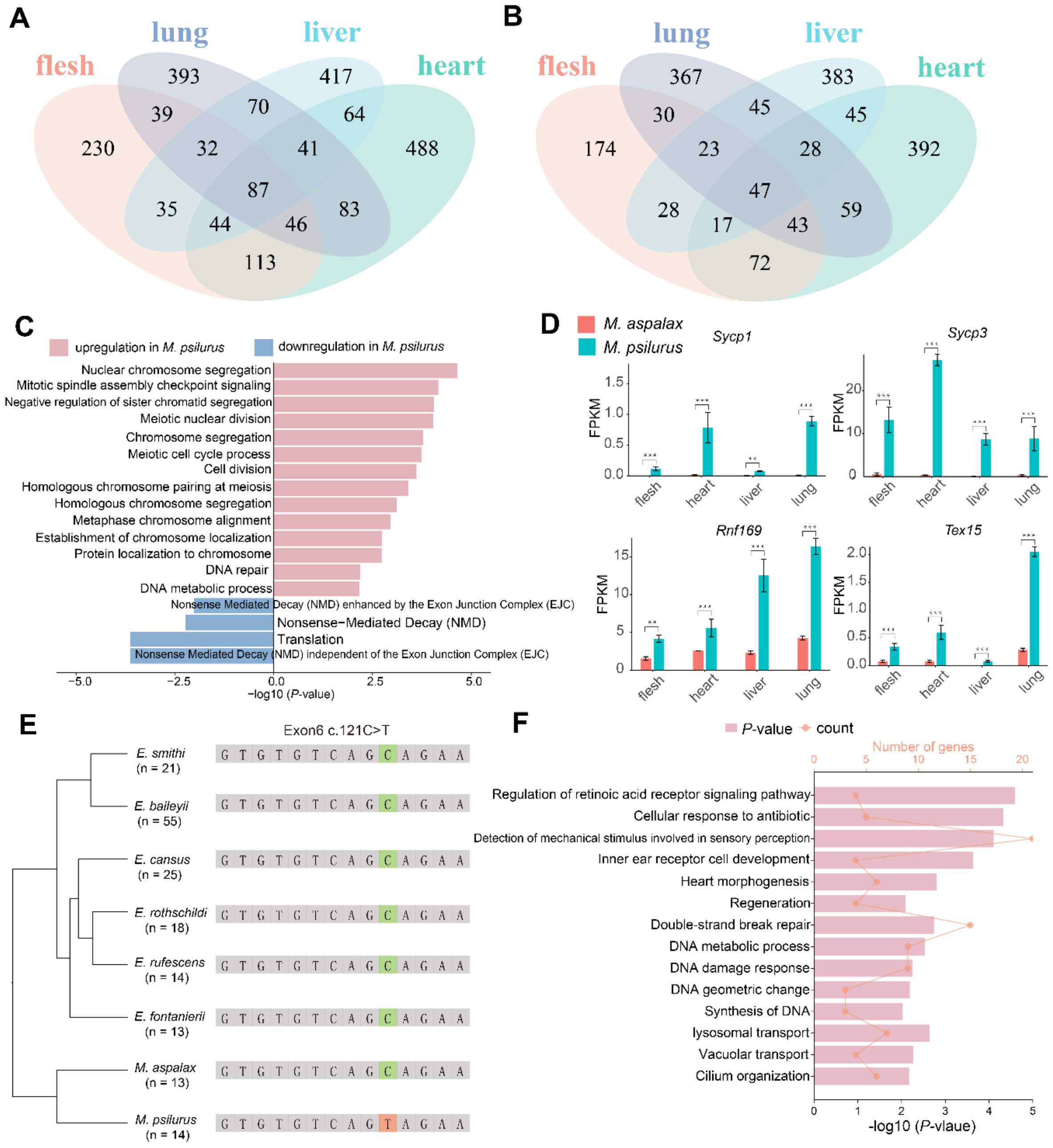

fig. S3.

Comparative transcriptomic and genetic analysis. (A) Venn diagram illustrates the number of genes with significantly downregulated expression ($\log_2$(fold change) > 1 and adjusted *P*-value < 0.005) in *M. psilurus* compared to *M. aspalax* across four tissues. **(B)** Venn diagram depicts the number of genes with significantly upregulated expression ($\log_2$(fold change) > 1 and adjusted *P*-value < 0.005) in *M. psilurus* compared to *M. aspalax* across four tissues. **(C)** Pathway enrichment analysis of genes commonly upregulated and downregulated in *M. psilurus* compared to *M. aspalax* across all four tissues. **(D)** Expression levels of four genes associated with the DNA repair pathway (as shown in C) in both species.

(E) The c.121C>T mutation in exon 6 of *Aplf* was confirmed in all 173 individuals across the eight species of Myospalacinae. **(F**) Significantly enriched functional pathway for related genes closest with fixed structure variants in *M. psilurus*. *** indicates a *P*-value less than 0.001, ** indicates a *P*-value less than 0.01, and * indicates a *P*-value less than 0.05.

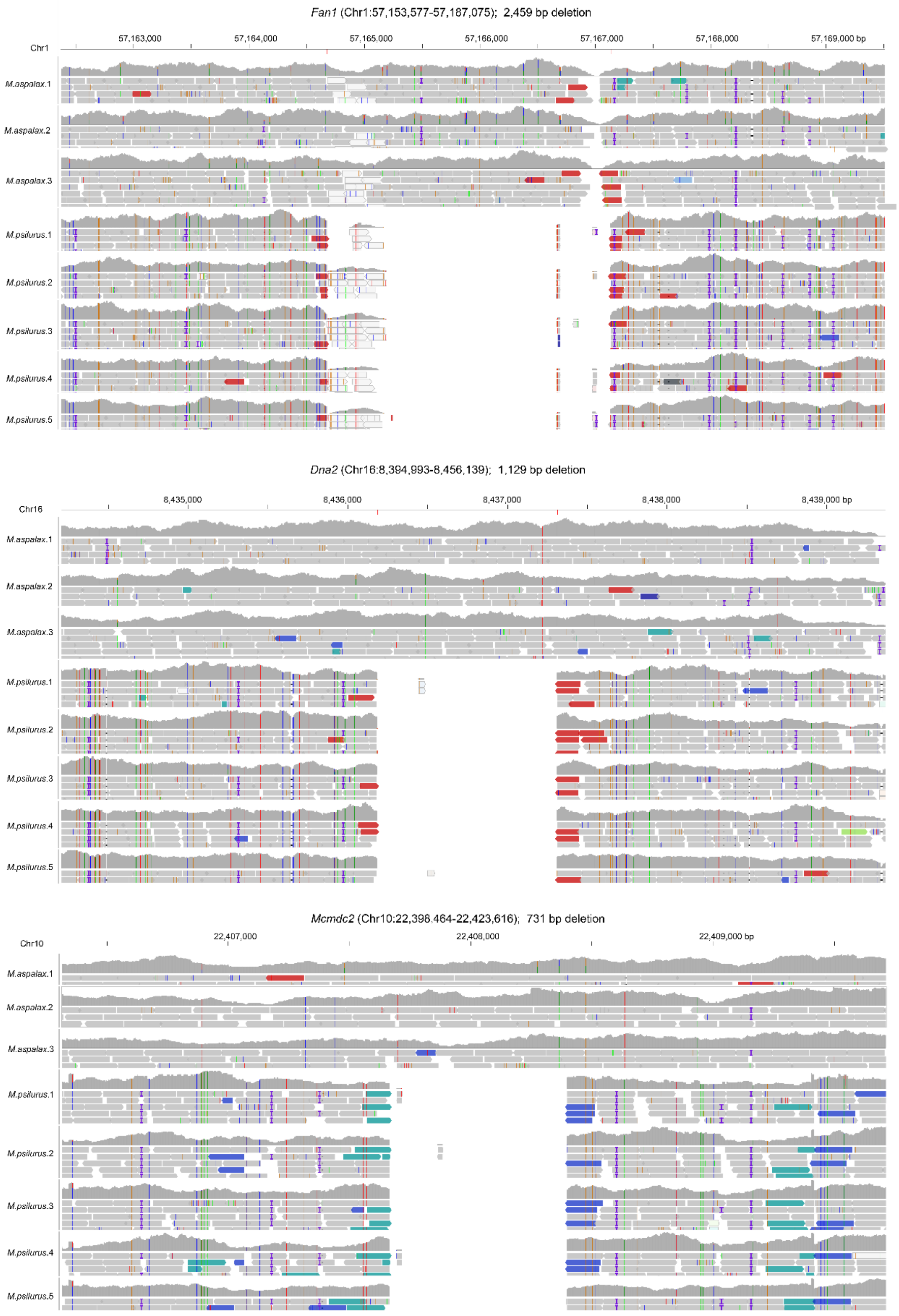

fig. S4.

IGV visualization for *M. psilurus*-specific SVs. Deletions specific to *M. psilurus* were identified in *Fan1*, Dna2, *Mcmdc2*, all of which are involved in DNA damage repair.

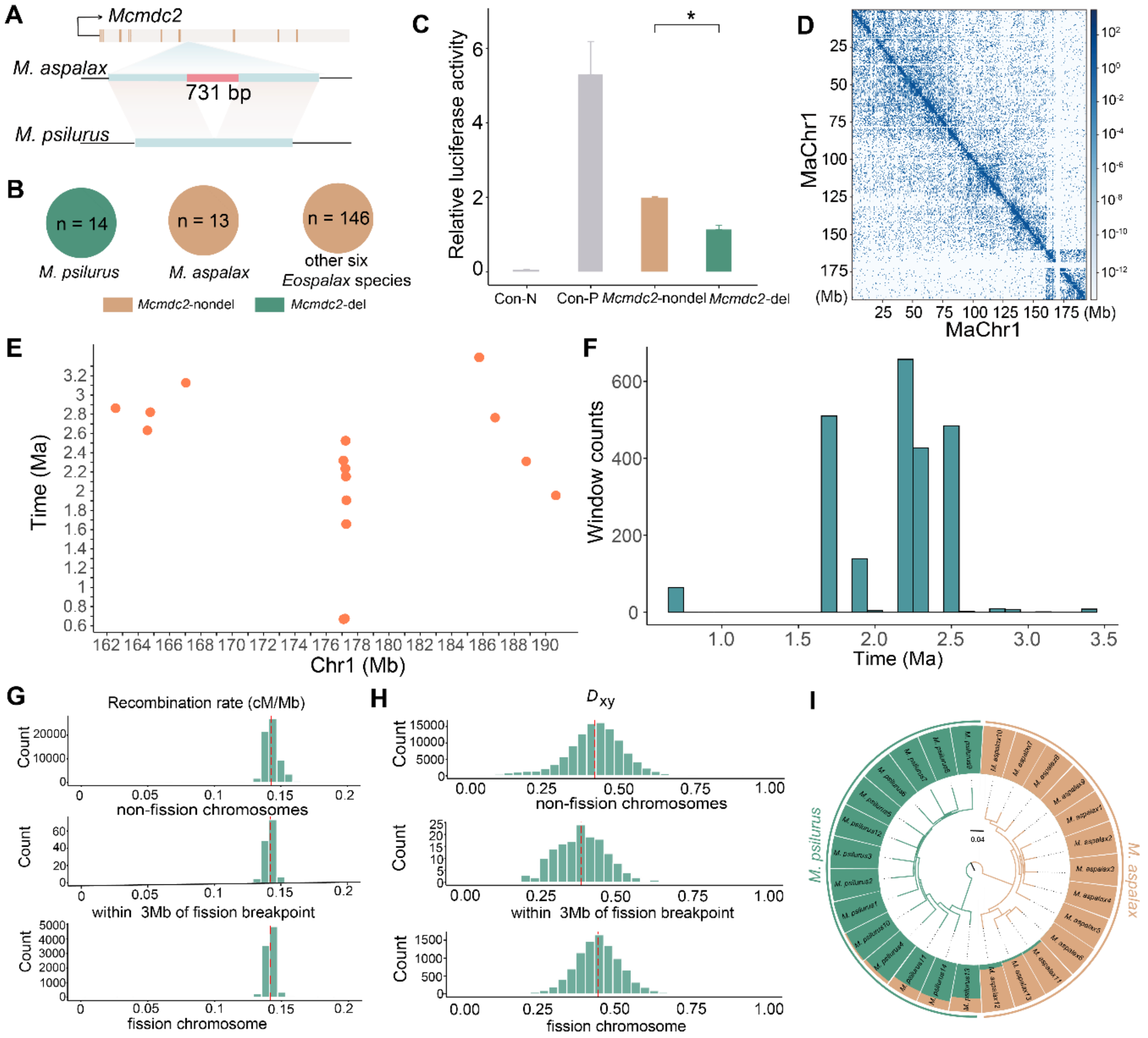

fig. S5.

Genomic structural variation of minichromosome maintenance domain containing 2 (*Mcmdc2*) in *M. psilurus* and impacts on chromosome fission. (A) A 731 bp deletion in an intron of *Mcmdc2* in *M. psilurus*. **(B)** Frequency of the *Mcmdc2* intronic deletion. All 14 *M. psilurus* samples exhibited the homozygous deletion, while it was absent in 159 samples of *M. aspalax* and six other *Eospslax* species. **(C)** Functional test of the *Mcmdc2* intronic deletion using a Dual-Luciferase Reporter Assay (DLRA) in H293T cells, showing decreased regulatory activity. pGL3-basic and pGL3-enhancer plasmids served as negative (Con-N) and positive controls (Con-P), respectively. **(D)** Hi-C interaction matrix for Chr1 and Chr32 of *M. psilurus* taking Chr1 of *M. aspalax* as reference. **(E)** Dot plot showing the divergence time between two species for selective SNPs in *M. psilurus* on the MaChr1b fragment (Chromosome 1 of *M. aspalax*: 160 Mb - 193.8 Mb, homologous to MpChr32) of the fission chromosome. **(F)** Frequency distribution histogram illustrating the window

counts for the range of divergence times for selective SNPs in **(E)**. **(G)** Recombination rate distribution across the two species on non-fissioned chromosomes, regions within 3 Mb of the fission breakpoint and fission chromosome, also with a window size of 40 kb. The x-axis indicates the range of recombination rate, and the y-axis represents the number of windows. The red dashed line marks the mean. **(H)** Distribution of D_{xy} between *M. aspalax* and *M. psilurus* on non-fissioned chromosomes, regions within 3 Mb of the fission breakpoint and fission chromosome and with the same window size. The x-axis shows D_{xy} range and the y-axis indicates the window counts. The red dashed line marks the mean. **(I)** Structure analysis indicated limited genetic admixture between two species.

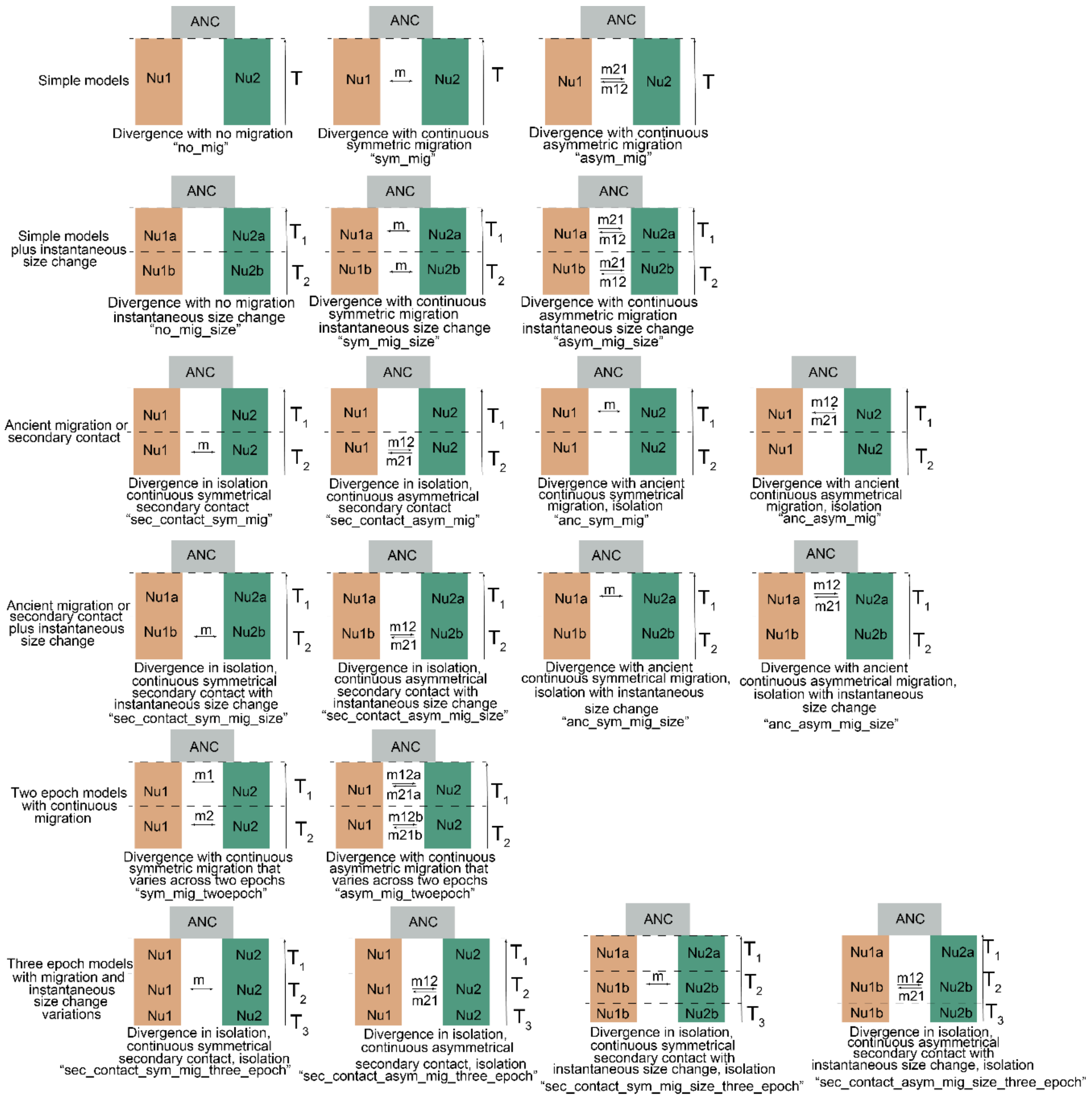

fig. S6.

Schematic for demographic scenarios modelled in ∂a∂i. In all models, an ancestral population diverged into two populations. The population sizes for *M. aspalax* and *M. psilurus* were showed in Nu1 and Nu2 respectively. T represents the generation time and m12 and m21 indicate the migration rate from *M. aspalax* to *M. psilurus* and from *M. psilurus* to *M. aspalax*.

table S1.

Statistics of sequencing data

Sequencing type	Species	Number of samples	Data information
HiFi	*M. aspalax*	1	table S2
	M. psilurus	1	table S3
HiC	*M. aspalax*	1	table S7
	M. psilurus	1	table S8
ChIP-Seq	*M. aspalax*	1	table S15
	M. psilurus	1	table S15
Bulk RNA-seq	*M. aspalax*	12	table S16
	M. psilurus	11	table S16
Iso-seq	*M. aspalax*	1	table S17
	M. psilurus	1	table S17
Long-read DNA sequencing	*M. aspalax*	2	table S18
	M. psilurus	3	table S18
Short-read DNA sequencing	*M. aspalax*	13	table S19
	M. psilurus	14	table S19
Short-read DNA sequencing	*M. myospalax*	1	table S20

table S2.

Statistics of long HiFi reads for genome assembly of *M. aspalax*

Sample	Base (Gbp)	Total number	Minimum Length (bp)	Average Length (bp)	Maximum Length (bp)	N50 (bp)
ccs1	30.82	1,952,288	48	15,787	49,786	15,790
ccs2	26.33	1,637,557	48	16,079	49,858	16,148
ccs3	26.72	1,719,114	49	15,545	50,033	15,600
Total	83.87	5,308,959	48	15,798	50,033	15,848

table S3.

Statistics of long HiFi reads for genome assembly of *M. psilurus*

Sample	Base (Gbp)	Total number	Minimum Length (bp)	Average Length (bp)	Maximum Length (bp)	N50 (bp)
ccs1	32.44	2,150,395	49	15,087	48,661	15,363
ccs2	32.59	2,215,511	48	14,710	46,344	14,897
ccs3	30.98	2,034,519	49	15,226	50,129	15,296
Total	96.01	6,400,425	48	15,000	50,129	15,179

table S4.

Assembly statistics of the two study genomes and genomes of closely related species

	**mMyoAsp6.1**	**mMyoPsi5.1**	***E. fontanierii*** **Liu et al (30)**	***E. baileyi*** **An et al (29)**
Sequencing platform	PacBio HiFi	PacBio HiFi	PacBio HiFi	ONT
Assembly level	Chromosome	Chromosome	Chromosome	Chromosome
Pseudo-chromosome number	31 ($2n$ = 62)	32 ($2n$ = 64)	31 ($2n$ = 62)	31 ($2n$ = 62)
Contig N50 (bp)	37,952,886	23,281,812	59,204,235	37,952,775
BUSCO completeness (%)	96.6	96.7	97.0	96.8
Protein coding gene number	21,060	21,004	21,656	21,642

table S5.

BUSCO assessment of the *M. aspalax* genome assembly

**Type**	**Number**	**Percentage (%)**
Complete BUSCOs (C)	8,915	96.60
Complete and single-copy BUSCOs (s)	8,801	95.40
Complete and duplicated BUSCOs (D)	114	1.20
Fragment BUSCOs (F)	58	0.60
Missing BUSCOs (M)	253	2.80
Total BUSCO groups searched	9,226	100

table S6.

BUSCO assessment of the *M. psilurus* genome assembly

**Type**	**Number**	**Percentage (%)**
Complete BUSCOs (C)	8,924	96.70
Complete and single-copy BUSCOs (s)	8,809	95.50
Complete and duplicated BUSCOs (D)	115	1.20
Fragment BUSCOs (F)	54	0.60
Missing BUSCOs (M)	248	2.70
Total BUSCO groups searched	9,226	100

table S7.

Statistics of Hi-C reads for *M. aspalax*

**Sample**	**Base (Gbp)**	**Reads number**	**Reads length (bp)**	**Q20 (%)**	**Q30 (%)**	**GC content (%)**
*M. aspalax*_1	149.05	496,838,486 x2	150;150	97.4;95.5	90.4;86.6	41.3;41.3
*M. aspalax*_2	163.01	543,371,345 x2	150;150	97.4;95.9	90.5;87.5	41.2;41.4

table S8.

Statistics of Hi-C reads for *M. psilurus*

Sample	Base (Gb)	Reads number	Reads length (bp)	Q20 (%)	Q30 (%)	GC content (%)
*M. psilurus*_1	153.77	512,558,258x2	150;150	97.4;96.1	90.5;88.0	41.4;41.5
*M. psilurus*_2	152.00	506,681,391x2	150;150	97.6;95.7	90.9;87.1	41.4;41.5

table S9.

Lengths of 31 pseudo-chromosomes for *M. aspalax*

Chromosome	Size (bp)
Chr1	193,890,730
Chr2	144,061,997
Chr3	140,519,636
Chr4	129,547,517
Chr5	126,510,743
Chr6	122,233,022
Chr7	113,777,412
Chr8	107,382,643
Chr9	99,495,847
Chr10	94,224,382
Chr11	93,344,036
Chr12	92,560,642
Chr13	89,298,488
Chr14	87,805,587
Chr15	87,139,734
Chr16	84,826,003
Chr17	79,425,235
Chr18	68,989,172
Chr19	65,851,678
Chr20	64,347,501
Chr21	63,143,761
Chr22	62,989,304
Chr23	61,476,047
Chr24	56,982,109
Chr25	55,719,797
Chr26	55,525,496
Chr27	53,100,550
Chr28	48,624,519
Chr29	46,999,249
Chr30	43,375,992
Chr31	42,859,516
Total	2,676,028,345

table S10.

Lengths of 32 pseudo-chromosomes for *M. psilurus*

Chromosome	Size (bp)
Chr1	164,292,605
Chr2	157,348,941
Chr3	141,444,018
Chr4	127,546,195
Chr5	114,404,836
Chr6	106,206,085
Chr7	105,257,458
Chr8	104,016,977
Chr9	98,264,258
Chr10	95,846,545
Chr11	93,003,916
Chr12	88,787,959
Chr13	84,296,945
Chr14	80,746,164
Chr15	79,096,405
Chr16	76,917,133
Chr17	75,749,456
Chr18	65,921,577
Chr19	64,512,086
Chr20	64,224,766
Chr21	63,483,942
Chr22	62,962,022
Chr23	59,597,249
Chr24	59,490,652
Chr25	55,667,820
Chr26	54,674,365
Chr27	51,979,650
Chr28	51,154,446
Chr29	47,472,031
Chr30	46,212,684
Chr31	45,878,234
Chr32	34,521,131
Total	2,620,978,551

table S11.

BUSCO assessment of the *M. aspalax* genome annotation

Type	Number	Percentage (%)
Complete BUSCOs (C)	8,924	92.4
Complete and single-copy BUSCOs (s)	8,809	91.4
Complete and duplicated BUSCOs (D)	115	1.0
Fragment BUSCOs (F)	54	1.6
Missing BUSCOs (M)	248	6.0
Total BUSCO groups searched	9,226	100

table S12.

BUSCO assessment of the *M. psilurus* genome annotation

Type	Number	Percentage (%)
Complete BUSCOs (C)	8,542	92.6
Complete and single-copy BUSCOs (s)	8,445	91.5
Complete and duplicated BUSCOs (D)	97	1.1
Fragment BUSCOs (F)	167	1.8
Missing BUSCOs (M)	517	5.6
Total BUSCO groups searched	9,226	100

table S13.

Repeats content of *M. aspalax* genome

Order		Length of sequence (bp)	Percentage of repeated sequence (%)	Percentage of whole genome sequence (%)
**DNA**		74,775,329	4.844	2.469
	DNA_CMC-EnSpm	9,788,134	0.634	0.323
	DNA_MuDR	1,247,418	0.081	0.041
	DNA_PIF-Harbinger	881,301	0.057	0.029
	DNA_hAT-Ac	2,371,409	0.154	0.078
	DNA_hAT-Tip100	4,949,623	0.321	0.163
	DNA_other	57,888,144	3.75	1.911
**LINE**		466,764,654	30.24	15.41
	LINE_L1	433,637,962	28.094	14.316
	LINE_L2	24,197,600	1.568	0.799
	LINE_other	9,340,370	0.605	0.308
**LTR**		438,521,250	28.41	14.477
	LTR_Copia	2,556,450	0.166	0.084
	LTR_Gypsy	50,294,852	3.258	1.66
	LTR_other	400,866,000	25.97	13.234
**Low_complexity**		44,483,164	2.882	1.469
**SINE**		461,380,410	29.891	15.232
**Satellite**		7,631,508	0.494	0.252
**Simple_repeat**		261,867,462	16.965	8.645
**Small_RNA**		1,760,251	0.114	0.058
**Unclassified**		89,143,692	0.048	0.029
**Total**		1,543,546,396		50.96

table S14.

Repeats content of *M. psilurus* genome

Order		Length of sequence (bp)	Percentage of repeated sequence (%)	Percentage of whole genome sequence (%)
DNA		66,463,055	3.562	2.006
	DNA_CMC-EnSpm	2,484,847	0.133	0.075
	DNA_MuDR	1,115,711	0.060	0.034
	DNA_PIF-Harbinger	552,176	0.030	0.017
	DNA_hAT-Ac	1,504,496	0.081	0.045
	DNA_hAT-Tip100	4,806,273	0.258	0.145
	DNA_other	57,539,697	3.084	1.737
LINE		425,925,645	22.826	12.855
	LINE_L1	397,794,945	21.319	12.006
	LINE_L2	22,626,541	1.213	0.683
	LINE_other	5,764,657	0.309	0.174
LTR		530,403,018	28.426	16.008
	LTR_Copia	14,797,135	0.793	0.447
	LTR_Gypsy	52,110,055	2.793	1.573
	LTR_other	482,094,879	25.837	14.55
Low_complexity		33,023,002	1.770	0.997
SINE		420,735,729	22.548	12.698
Satellite		8,439,720	0.452	0.255
Simple_repeat		611,842,786	32.790	18.466
Small_RNA		4,336,748	0.232	0.131
Unclassified		104,946,083	0.048	0.032
Total		1,865,928,741		56.31

table S15.

Summary statistics of ChIP-Seq reads

Sample Name	Raw pairs num	Raw bases (Gb)	Clean pairs num	Clean bases (Gb)	Clean rate (%)	Q20 (%)	Q30 (%)	GC (%)
M. psilurus	19,058,756	5.72	18,960,924	4.5	99.49	98.21	94.49	44.9
M. aspalax	25,941,767	7.78	25,729,787	5.39	99.18	98.29	94.59	43.82

table S16.

Statistics of Bulk RNA sequencing

Sample	Tissue	Paired_end	Total Reads	Total Bases (bp)	Q20	Q30
*M. aspalax*1	Fresh	R1	89,750,943	13,152,652,804	98.08%	94.69%
*M. aspalax*1	Fresh	R2	89,750,943	13,146,243,208	98.10%	94.68%
*M. aspalax*1	Heart	R1	33,410,495	4,931,620,345	98.01%	94.38%
*M. aspalax*1	Heart	R2	33,410,495	4,930,001,074	97.72%	93.64%
*M. aspalax*1	Liver	R1	71,004,435	10,554,198,385	98.33%	95.12%
*M. aspalax*1	Liver	R2	71,004,435	10,549,862,283	98.07%	94.42%
*M. aspalax*1	Lung	R1	61,945,025	9,212,481,116	98.30%	95.04%
*M. aspalax*1	Lung	R2	61,945,025	9,211,577,420	97.94%	94.06%
*M. aspalax*2	Fresh	R1	40,690,668	6,028,815,748	98.25%	94.97%
*M. aspalax*2	Fresh	R2	40,690,668	6,022,558,159	97.61%	93.37%
*M. aspalax*2	Heart	R1	76,170,729	11,274,147,256	97.92%	94.32%
*M. aspalax*2	Heart	R2	76,170,729	11,268,710,192	98.02%	94.41%
*M. aspalax*2	Liver	R1	49,954,488	7,427,105,882	98.37%	95.25%
*M. aspalax*2	Liver	R2	49,954,488	7,419,356,322	97.46%	92.93%
*M. aspalax*4	Fresh	R1	51,126,896	7,591,605,000	98.34%	95.20%
*M. aspalax*4	Fresh	R2	51,126,896	7,583,062,607	97.50%	93.07%
*M. aspalax*4	Lung	R1	45,464,140	6,759,826,646	98.40%	95.34%
*M. aspalax*4	Lung	R2	45,464,140	6,754,493,389	97.43%	92.93%
*M. aspalax*6	Fresh	R1	28,716,442	4,256,888,150	98.25%	93.89%
*M. aspalax*6	Fresh	R2	28,716,442	4,223,954,773	96.19%	89.18%
*M. aspalax*6	Liver	R1	40,306,795	5,992,153,116	97.98%	93.10%
*M. aspalax*6	Liver	R2	40,306,795	5,942,688,556	95.88%	88.32%
*M. aspalax*6	Lung	R1	43,915,904	6,510,590,936	98.05%	93.33%
*M. aspalax*6	Lung	R2	43,915,904	6,455,548,692	95.66%	87.89%
*M. psilurus*5	Fresh	R1	29,726,848	4,398,764,330	97.86%	92.71%
*M. psilurus*5	Fresh	R2	29,726,848	4,363,421,885	95.85%	88.33%
*M. psilurus*5	Liver	R1	35,618,480	5,286,913,048	98.05%	93.31%
*M. psilurus*5	Liver	R2	35,618,480	5,244,200,698	95.96%	88.55%
*M. psilurus*5	Lung	R1	33,948,239	5,038,529,658	97.72%	92.31%
*M. psilurus*5	Lung	R2	33,948,239	4,986,005,825	95.19%	86.60%
*M. psilurus*6	Fresh	R1	43,825,552	6,475,440,539	98.19%	94.90%
*M. psilurus*6	Fresh	R2	43,825,552	6,474,099,996	97.65%	93.47%
*M. psilurus*6	Heart	R1	57,063,726	8,441,249,052	98.24%	94.99%
*M. psilurus*6	Heart	R2	57,063,726	8,438,849,286	97.63%	93.38%
*M. psilurus*6	Liver	R1	55,231,283	8,186,673,301	98.12%	94.58%
*M. psilurus*6	Liver	R2	55,231,283	8,181,982,503	97.42%	92.85%
*M. psilurus*6	Lung	R1	46,630,121	6,923,239,848	98.48%	95.64%
*M. psilurus*6	Lung	R2	46,630,121	6,916,473,615	97.31%	92.64%
*M. psilurus*7	Fresh	R1	51,832,187	7,677,675,677	98.29%	95.13%
*M. psilurus*7	Fresh	R2	51,832,187	7,674,862,209	97.42%	92.84%

Sample	Tissue	Paired_end	Total Reads	Total Bases (bp)	Q20	Q30
*M. psilurus*7	Heart	R1	58,226,889	8,667,572,560	98.09%	94.46%
*M. psilurus*7	Heart	R2	58,226,889	8,659,943,970	97.55%	93.17%
*M. psilurus*7	Liver	R1	53,364,699	7,906,650,737	98.35%	95.29%
*M. psilurus*7	Liver	R2	53,364,699	7,901,514,927	97.49%	93.04%
*M. psilurus*7	Lung	R1	41,919,453	6,223,190,798	98.22%	94.92%
*M. psilurus*7	Lung	R2	41,919,453	6,218,059,984	97.17%	92.24%

table S17.

Statistics of isoform sequencing data

Sample	Total Reads	Total Bases (bp)
M. aspalax	3,422,492	5,106,718,751
M. psilurus	3,217,170	4,997,326,696

table S18.

Statistics of long-read DNA sequencing

Sample	Total Reads	Total Bases (bp)
M. aspalax.2	1,547,041	30,723,495,487
M. aspalax.8	1,118,899	22,054,539,700
M. psilurus.1	1,444,916	27,173,662,425
M. psilurus.5	1,841,102	32,735,721,715
M. psilurus.8	1,712,787	29,913,281,572

table S19.

Statistics of short-read DNA sequencing for *M. aspalax* and *M. psilurus*

Sample	Clean_bases (bp)	Mapping Rate	Mean Depth	Coverage
*M. aspalax*1	60,293,232,498	99.95%	19.65	98.20%
*M. aspalax*2	57,921,062,984	99.96%	18.88	98.30%
*M. aspalax*3	65,841,130,464	99.96%	21.47	98.80%
*M. aspalax*4	79,203,446,352	99.96%	25.83	98.54%
*M. aspalax*5	50,817,268,574	99.95%	16.56	98.75%
*M. aspalax*6	154,832,414,898	99.96%	49.2	99.47%
*M. aspalax*7	53,592,143,628	99.95%	17.46	98.62%
*M. aspalax*8	66,115,951,076	99.93%	21.58	98.54%
*M. aspalax*9	61,021,426,550	99.93%	19.89	99.40%
*M. aspalax*10	63,069,948,346	99.93%	20.58	99.39%
*M. aspalax*11	64,463,562,588	99.92%	21.01	99.30%
*M. aspalax*12	59,194,512,412	99.95%	19.31	98.28%
M. aspalax.13	59,194,749,790	99.92%	21.88	99.24%
*M. psilurus*1	49,493,752,812	99.59%	15.8	93.91%
*M. psilurus*2	50,997,401,792	99.52%	16.32	93.63%
*M. psilurus*3	68,745,964,906	99.61%	21.98	94.33%
*M. psilurus*4	50,381,139,702	99.58%	16.1	93.67%
*M. psilurus*5	154,832,414,898	99.26%	49.37	95.28%
*M. psilurus*6	61,806,597,666	99.61%	19.76	93.87%
*M. psilurus*7	64,593,724,956	99.60%	20.66	94.02%
*M. psilurus*8	50,159,959,038	99.63%	16.06	93.75%
*M. psilurus*9	60,191,064,044	99.57%	19.25	94.01%
*M. psilurus*10	66,513,866,936	99.44%	21.13	95.10%
*M. psilurus*11	58,687,122,570	99.46%	18.62	94.68%
*M. psilurus*12	60,154,827,380	97.91%	18.8	94.67%
*M. psilurus*13	57,106,633,286	99.44%	18.09	93.05%
*M. psilurus*14	65,098,949,386	99.41%	20.65	94.22%

table S20.

Statistics of short-read DNA sequencing for *M. myospalax*

Sample	Total Reads	Total Bases (bp)	Read Length	Q20 (%)	Q30 (%)	GC (%)
M. myospalax	6,323,388	1,264,677,600	PE100	97.00	90.46	43.85

table S21.

Primers for validating isoforms

Targets	Primer Fw	Primer Rev
Aplf	TCCTTCCTAGGCGAATGTGTAGAC	CTGTAGCAGTTTGCCCCATACATG
β-actin	CATCACACCTTCTACAACGAGC	CATCGCCGGAGTCCATCACAA

table S22.

Primers for CRISPR-Cas9

Targets	Primer Fw	Primer Rev
sgRNA_1	CACCGGTCTTCACAATATCCACATC	AAACGATGTGGATATTGTGAAGAC
sgRNA_2	CACCGCATAGTAAAGGACCCAGATG	AAACCATCTGGGTCCTTTACTATG
sgRNA_3	CACCGGAAGGACGGGATTTTAATAG	AAACCTATTAAAATCCCGTCCTTC
sgRNA_4	CACCGCGGAGGACGGCCTGCATGTA	AAACTACATGCAGGCCGTCCTCCG
sgRNA_5	CACCGGGAGGACGGCCTGCATGTAT	AAACATACATGCAGGCCGTCCTCC
sgRNA_6	CACCGCATACATGCAGGCCGTCCTC	AAACGAGGACGGCCTGCATGTATG

Data S1. (separate file)

Climate factors (bio1~bio19) of all sampling sites

Data S2. (separate file)

Estimated demographic populations for all models of ∂a∂i
